# From metabolic network to brain loop: unraveling the neuro-metabolic correlates in PCOS-related depression

**DOI:** 10.3389/fendo.2026.1925127

**Published:** 2026-09-21

**Authors:** Yi Cao, Zhaoying Li, Yuxing Zhang, Qian Wen, Yuanyuan Shi, Jing Xie, Dongfeng Zhang

**Affiliations:** 1 Women and Children’s Hospital, Qingdao University, Qingdao, Shandong, China; 2 Biomedical Center of Qingdao University, Qingdao, Shandong, China; 3 Department of Epidemiology and Health Statistics, Public Health College, Qingdao University, Qingdao, Shandong, China

**Keywords:** PCOS, depression, neuro-metabolism, BMI, hyperandrogenism, inflammation, insulin resistance, neuroimaging

## Abstract

**Aim:**

Polycystic ovary syndrome(PCOS) is increasingly recognized for its substantial depressive burden, however, the underlying mechanism remains unclear. In this study, the neuro-metabolic pathway was explored to unravel the mechanism in PCOS-related depression.

**Methods:**

Participants recruited from the UK Biobank comprised a cross-sectional population, the outcome as female depression, with 2,826 individuals among whom 471 diagnosed with PCOS. Progressive regression, interaction and mediation analyses, linearity and threshold examinations, relative weight analysis and principal component analysis(PCA), as well as stratified analyses were conducted in this study.

**Results:**

The two brain magnetic resonance imaging(MRI) metrics as volume of grey matter in frontal orbital cortex(FOC GMVol) and mean thickness of caudalanteriorcingulate(CaudalAntCing Thk) showed prominent links with PCOS-related depression, with CaudalAntCing Thk serving as the final common executor to depression. FOC GMVol and CaudalAntCing Thk interacted with PCOS in a metabolic-dependent and a metabolic-independent way respectively. A PCOS×free androgen index(FAI)×FOC GMVol three-way interaction was observer only in normal body mass index(BMI) individuals with high FAI. The FOC GMVol-CaudalAntCing Thk association, correlated with concurrent increases in BMI and FAI, existed exclusively in non-PCOS. Metabolic factors exhibited mediating effects (BMI and FAI) and formed a network among which BMI served as a hub linking the antagonistic FAI and the auxiliary triglyceride-glucose index(TyG), while systemic immune-inflammation index(SII) acted as an independent risk factor irrelevant to PCOS. A two-tiered brain protective hypothesis was proposed against depression: neural compensation reflected the protective FOC GMVol-CaudalAntCing Thk coupling in non-PCOS, and protective threshold patterns of both FOC GMVol and CaudalAntCing Thk in PCOS, with FAI playing pivotal and distinct roles at both peripheral and central levels across the entire pathways.

**Conclusion:**

Given the intricate neuro-metabolic correlates underlying PCOS-related depression, further studies were warranted and a synergistic therapeutic strategy of androgen suppression plus weight management might be considered.

## Introduction

Polycystic ovary syndrome(PCOS) is one of the most common endocrine and metabolic disorders in reproductive-aged women, affecting approximately 10-13% of this population worldwide (1). Beyond the well-established reproductive and metabolic consequences, PCOS is increasingly recognized for its substantial psychiatric burden (2, 3). Epidemiological studies demonstrate that women with PCOS exhibit a increasing prevalence of depression(36.6%) compared with their non-PCOS counterparts(14.2%), making the overall probability of depression in PCOS patients 2.5-fold higher (4–6). This elevated risk impairs quality of life and complicates clinical management in PCOS patients.

Hyperandrogenism, obesity, insulin resistance and low-grade chronic inflammation in PCOS have been considered potential risks of the PCOS-depression association (7–9). Emerging evidences suggest that brain regions involved in emotion regulation play key roles in depression pathophysiology (10, 11) and these regions are sensitive to metabolic signals such as insulin, adipokines and sex hormones, suggesting a potential metabolic-brain axis (12–14). In PCOS, metabolic disturbances disrupt the structural and functional integrity of brain regions, thereby increasing depression vulnerability (15, 16). Thus, existing literature supports a metabolic-dominant pathway, in which peripheral metabolic dysregulation serves as the primary driver of PCOS-related depression, while brain structural alterations are regarded as secondary, metabolic-induced changes. However, the possibility remains that brain structural alterations may exhibit independent associations with depression susceptibility or exhibit larger effects than peripheral metabolic dysfunction. The specific contributions of brain structural metrics and their interplay with metabolic pathways in PCOS-related depression remain unclear.

Addressing this question requires an integrative approach considering metabolic networks, brain structural profiles and their interplay with PCOS status. In this study, we systematically explored the metabolic and brain structural pathways underlying PCOS-related depression using data from the UK Biobank. We sought to: (1) identify the respective correlational contributions of metabolic biomarkers and brain magnetic resonance imaging(MRI) metrics to the PCOS-depression association; (2) examine the interrelationships among metabolic biomarkers and among brain metrics; and (3) characterize the interactions between metabolic and brain structural factors. By addressing these objectives, we aimed to provide novel insights into the neuro-metabolic correlates linking PCOS to depression.

## Methods

### Study population

The UK Biobank is a prospective cohort study that recruited over 500,000 participants aged 40 to 69 years from 2006 to 2010, with massive data collection covering baseline demographics, lifestyle factors, diagnoses, medications, and multiorgan MRI data. All participants were with written informed consent, and the ethical approval was granted by North West Multi-Centre Ethics Committee. Following the study design(**Supplementary Figure 1**), after excluding hypertension, diabetes, hypothyroidism, hyperprolactinemia and adrenal enzyme deficiency from female participants(diagnostic criteria: ICD-9/10, self-report at recruitment, or systolic blood pressure ≧ 140 mmHg/diastolic blood pressure ≧ 90 mmHg for blood pressure, or HbA1c ≧ 48 mmol/mol for diabetes), 471 PCOS cases(ICD-9/10 or self-report at recruitment) were identified and matched to non-PCOS female controls(1:5 ratio) using propensity score matching by age(caliper = 0.2), yielding a cross-sectional sample of 2,826 women. The outcome of interest was female depression (ICD-9/10 or self-report at recruitment).

### Basic, metabolic, and brain covariates

Information including age at recruitment, ethnicity, education level, physical activity, smoking status, alcohol consumption and menopause status was treated as basic covariates. Testosterone, sex hormone-binding globulin(SHBG), platelet, lymphocyte, neutrophil, triglycerides and glucose were extracted from the blood samples collected at recruitment. Body mass index(BMI), free androgen index(FAI), systemic immune-inflammation index(SII) and triglyceride-glucose index(TyG) were calculated as metabolic biomarkers with the formulas and clinical cut-offs provided as below:


$$\text{BMI}\;=\;\text{weight}\;(\text{kg})\;/\;{\left[\text{height}\;(\text{m})\right]}^{2},\;\text{normal}\;\text{weight}\;<25.0\;\text{kg}/{\text{m}}^{2},\;\text{overweight}\;25.0\;\sim \;29.9\text{kg}/{\text{m}}^{2},\;\text{obesity}\;≧\;30.0\;\text{kg}/{\text{m}}^{2}.$$

FAI = [total testosterone (nmol/L)/SHBG (nmol/L)]×100,normoandrogenism≦ 5, hyperandrogenism> 5.

$$\text{SII}\;=\;[\text{platelet}\;\text{count}\;(\times 10^{9}/\text{L})\times \text{neutrophil}\;\text{count}\;(\times 10^{9}/\text{L})]/\text{lymphocyte}\;\text{count}\;(\times 10^{9}/\text{L}),\;\text{low}\;\text{inflammation}<\;500,\;\text{high}\;\text{inflammation}≧\;500.$$

TyG = Ln [fasting triglycerides (mg/dL)×fasting glucose (mg/dL)/2], non−insulin resistance < 8.5, insulin resistance≧ 8.5.

Brain covariates were derived from 3.0T magnetic resonance imaging(MRI) scans processed with standard imaging pipelines in UK Biobank. Based on previous evidence linking these metrics to PCOS, we focused on four categories(detailed in **Table 1**): (i) regional brain volume, (ii) cortical thickness, (iii) diffusion MRI(dMRI) metrics including fractional anisotropy (FA) and intravoxel incoherent motion (ICVF) and (iv) task-functional MRI(fMRI) metrics. After averaging left and right hemispheres, hierarchical filtering and residual correction were applied to regional brain volume, cortical thickness, and dMRI metrics to remove confounders. Linear regression was used to adjust for total intracranial volume (regional volume), global mean thickness(cortical thickness), and global mean FA/ICVF and mean head motion(dMRI metrics). For task-fMRI, absolute head motion was included as a covariate.

**Table 1:** Baseline characteristics of the study population.

Variable	All (n = 2826) Median (IQR)/number (%)	With PCOS (n = 471) Median (IQR)/number (%)	Without PCOS (n =2355) Median (IQR)/number (%)	p-value
Basic covariates				
Age at recruitment	45 (42, 49)	45 (42, 49)	45 (42, 49)	1.00
Ethnicity				
Non-White	219 (7.75%)	49 (10.40%)	170 (7.22%)	0.02 *
White	2607 (92.25%)	422 (89.60%)	2185 (92.78%)	
Education level				
Basic_or_none	164 (5.80%)	12 (2.55%)	152 (6.45%)	<0.001 **
University	1175 (41.58%)	230 (48.83%)	945 (40.13%)	
Secondary	1450 (51.31%)	223 (47.35%)	1227 (52.10%)	
Physical activity				
Inactive	175 (6.19%)	33 (7.01%)	142 (6.03%)	0.40
Light	2166 (76.65%)	357 (75.80%)	1809 (76.81%)	
Moderate	431 (15.25%)	74 (15.71%)	357 (15.16%)	
Vigorous	11 (0.39%)	0 (0.00%)	11 (0.47%)	
Smoking status				
Never	1724 (61.00%)	293 (62.21%)	1431 (60.76%)	0.43
Previous	770 (27.25%)	133 (28.24%)	637 (27.05%)	
Current	320 (11.32%)	43 (9.13%)	277 (11.76%)	
Alcohol consumption				
Never	127 (4.49%)	26 (5.52%)	101 (4.29%)	0.01 **
Previous	86 (3.04%)	25 (5.31%)	61 (2.59%)	
Current	2605 (92.18%)	419 (88.96%)	2186 (92.82%)	
Menopause status				
No	1903 (67.34%)	313 (66.45%)	1590 (67.51%)	<0.001 **
Yes	555 (19.64%)	74 (15.71%)	481 (20.43%)	
Not sure	356 (12.59%)	81 (17.20%)	275 (11.68%)	
Metabolic biomarkers				
BMI	24.92 (22.5, 28.01)	26.15 (23.12, 30)	24.62 (22.41, 27.61)	<0.001 **
FAI	1.61 (0.93, 2.64)	1.96 (1.02, 3.36)	1.56 (0.91, 2.53)	<0.001 **
SII	556.04 (415.44, 749.85)	578.78 (434.81, 748.22)	552.96 (411.07, 751.3)	0.17
TyG	8.32 (8.02, 8.69)	8.39 (8.12, 8.74)	8.3 (8, 8.67)	<0.001 **
Brain MRI metrics				
Regional brain volume				
Hippocampus GMVol	-0.03 (-0.56, 0.47)	0.01 (-0.61, 0.51)	-0.05 (-0.53, 0.46)	0.97
Amygdala GMVol	-0.02 (-0.5, 0.45)	-0.16 (-0.53, 0.47)	0 (-0.48, 0.4)	0.85
FMC GMVol	-0.04 (-0.69, 0.55)	-0.16 (-0.68, 0.46)	-0.03 (-0.69, 0.57)	0.80
FOC GMVol	-0.03 (-0.5, 0.45)	-0.01 (-0.53, 0.33)	-0.03 (-0.49, 0.46)	0.47
Accumbens Vol	0.04 (-0.54, 0.54)	-0.05 (-0.73, 0.6)	0.07 (-0.46, 0.52)	0.40
Thalamus Vol	-0.02 (-0.42, 0.39)	-0.03 (-0.45, 0.48)	-0.02 (-0.41, 0.36)	0.97
Cortical thickness				
RostAntCing Thk	0.01 (-0.1, 0.11)	0.02 (-0.11, 0.14)	0.01 (-0.1, 0.09)	0.32
CaudalAntCing Thk	0.01 (-0.16, 0.18)	0.04 (-0.15, 0.14)	0.01 (-0.16, 0.18)	0.90
Insula Thk	0.01 (-0.09, 0.1)	0 (-0.09, 0.11)	0.01 (-0.08, 0.09)	0.99
MedOrbFront Thk	0.01 (-0.08, 0.1)	0.01 (-0.06, 0.11)	0.01 (-0.09, 0.09)	0.35
LatOrbFront Thk	0 (-0.08, 0.09)	0 (-0.06, 0.08)	0 (-0.08, 0.09)	0.73
dMRI metrics				
CH FA	0.46 (0.44, 0.48)	0.46 (0.45, 0.48)	0.46 (0.44, 0.48)	0.83
CH ICVF	0.5 (0.48, 0.52)	0.5 (0.48, 0.51)	0.5 (0.48, 0.52)	0.20
CCG FA	0.61 (0.59, 0.63)	0.61 (0.59, 0.62)	0.61 (0.59, 0.63)	0.48
CCG ICVF	0.61 (0.58, 0.63)	0.6 (0.58, 0.63)	0.61 (0.59, 0.63)	0.25
UF FA	0.52 (0.5, 0.54)	0.51 (0.5, 0.54)	0.52 (0.5, 0.54)	0.16
UF ICVF	0.52 (0.5, 0.53)	0.51 (0.5, 0.53)	0.52 (0.5, 0.53)	0.17
Task-based fMRI				
Amygdala BOLD Effect	0.37 (0.3, 0.47)	0.36 (0.29, 0.43)	0.37 (0.3, 0.48)	0.31
Outcome variable				
Non-depression	2443 (86.45%)	376 (79.83%)	2067 (87.77%)	<0.001 **
Depression	383 (13.55%)	95 (20.17%)	288 (12.23%)	

Baseline characteristics were presented as proportions for categorical variables and as medians with IQRs for continuous variables. Between-group comparisons were conducted using the t-test or Wilcoxon rank-sum test for continuous data and the χ²test for categorical data. *p-value<0.05, **p-value<0.01 were considered statistically significant.

“Prefer not to answer” categories for Smoking (n=6), Alcohol.drinker (n=2) and menopause (n=12) were excluded from the table due to extremely small sample size but were included in all statistical calculations.

Hippocampus GMVol: volume of grey matter in hippocampus.

Amygdala GMVol: volume of grey matter in amygdala.

FMC GMVol: volume of grey matter in frontal medial cortex; frontal medial cortex corresponded to medial prefrontal cortex (mPFC).

FOC GMVol: volume of grey matter in frontal orbital cortex; frontal orbital cortex corresponded to orbitofrontal cortex (OFC).

Accumbens Vol: volume of accumbens.

Thalamus Vol: volume of thalamus.

RostAntCing Thk: mean thickness of rostralanteriorcingulate; rostralanteriorcingulate corresponded to rostral anterior cingulate cortex (rostral ACC).

CaudalAntCing Thk: mean thickness of caudalanteriorcingulate; caudalanteriorcingulate corresponded to caudal anterior cingulate cortex (caudal ACC).

Insula Thk: mean thickness of insula.

MedOrbFront Thk: mean thickness of medialorbitofrontal.

LatOrbFront Thk: mean thickness of lateralorbitofrontal.

CH FA: mean FA in cingulum hippocampus on FA skeleton;

CH ICVF: mean ICVF in cingulum hippocampus on FA skeleton.

CCG FA: mean FA in cingulum cingulate gyrus on FA skeleton.

CCG ICVF: mean ICVF in cingulum cingulate gyrus on FA skeleton.

UF FA: mean FA in uncinate fasciculus on FA skeleton;

UF ICVF: mean ICVF in uncinate fasciculus on FA skeleton;

Amygdala BOLD Effect: 90th percentile of blood oxygen level-dependent effect (in group-defined amygdala activation mask) for faces-shapes contrast.

### Descriptive statistics

Baseline characteristics were presented as proportions for categorical variables and as medians with interquartile ranges(IQRs) for continuous variables. Between-group comparisons were conducted using the t-test or Wilcoxon rank-sum test for continuous data and the χ²test for categorical data.

### Logistic regression analyses

Conditional logistic regression models were constructed to examine the associations of PCOS and metabolic biomarkers with depression: Model 1(crude), Model 2(adjusted for basic covariates) and Model 3(further adjusted for metabolic biomarkers). To evaluate the associations involving brain metrics(smaller sample size), Bayesian logistic regression models were implemented: Model 4(all brain metrics), Model 5(metabolic model), Model 6(specific brain metrics) and Model 7(metabolic biomarkers plus specific brain metrics).

### Interaction analyses and mediation analyses

Multiplicative and additive interaction analyses were performed for metabolic biomarkers based on Model 3. For brain metrics, multiplicative interactions(PCOS×z-scored brain metrics) were estimated in Model 4 using Bayesian inference with Hamiltonian Monte Carlo sampling implemented in Stan.

In mediation analyses, structural equation modeling(SEM) with bias-corrected bootstrapping(5,000 resamples) was applied to metabolic biomarkers based on Model 3. Bayesian mediation analyses were conducted for brain metrics within the Model 4.

### Relative weight analysis and PCA analysis

Relative weight analysis using the LMG(Lindeman, Merenda, and Gold) method was performed to quantify the contributions of metabolic biomarkers, brain metrics and PCOS status to depression. PCA was conducted to explore the underlying correlation structures. Weighted linear regression was used instead of Bayesian models due to non-convergence in the complex covariate-rich environment.

### Linearity and threshold analyses

To metabolic biomarkers, grouped and continuous analyses were performed based on Model 3. In the grouped analysis, metabolic biomarkers were categorized according to clinical cut-offs, with the lowest category as the reference. In the continuous analysis, generalized additive models (GAMs) with a logit link function and smoothing splines were used to examine non-linear trends. Associations were assessed using the χ²test; effective degrees of freedom (edf) approximating 1 indicated linearity.

For brain metrics, linear and quadratic regression models with PCOS×brain metric² interaction terms were constructed in PCOS, adjusting for age, ethnicity and BMI. Model fit (linear vs. quadratic) was compared via ANOVA, and Johnson-Neyman analysis identified threshold intervals. In non-PCOS, linear and quadratic regression models tested brain metric-depression associations with the same covariates, and segmented regression detected threshold inflection points. For each metric, participants were stratified into inside- vs. outside-threshold subgroups; t-tests compared brain metrics between PCOS and non-PCOS within each subgroup.

### Stratified analyses to brain metric

PCOS/non-PCOS stratified analyses with and without metabolic adjustment were performed using Bayesian and stratified logistic regression. Simple slope analyses on the Logit scale interpreted brain metric-depression associations within each subgroup, and PCOS×brain metric interaction were tested via ANOVA.

Metabolic stratified analyses were conducted using weighted linear regression, adjusting for other metabolic biomarkers and basic covariates. Clinical cut-offs were applied for BMI, SII, and TyG; FAI was median-split. Among normal-BMI participants, further stratification compared high- vs. low-FAI subgroups.

### Stratified analyses to FOC GMVol-CaudalAntCing Thk association

To examine the relationship between FOC GMVol and CaudalAntCing Thk, interaction and mediation analyses were conducted across PCOS/non-PCOS subgroups using weighted linear regression, with and without metabolic adjustment. Stratified mediation analyses by PCOS and metabolic biomarkers were performed to the FOC GMVol-CaudalAntCing Thk association.

### Statistical analysis

Data were presented as odds ratio(OR)/β or bootstrap β with 95% confidence interval(CI)/credible interval(CrI)/highest density intervals(HDIs). * p-value<0.05, probability of direction(Pd) >97.5%, ** p-value<0.01, Pd >99.5% were considered statistically significant. The significance was also determined by 95% CI/CrI/HDI excluded zero. † Marginal trends were defined as p-value closed to 0.05 or 95% ≦ Pd< 97.5%, indicating tendency without statistical robustness. Analyses were performed using R version 4.5.1(2025-06-13).

## Results

### Participant characteristics

The baseline characteristics were presented in **Table 1**. Compared with non-PCOS, PCOS individuals exhibited a higher prevalence of depression, as well as elevated BMI, FAI, and TyG. No significance in SII or brain metrics was observed between the two groups.

### Prominent role of CaudalAntCing Thk in logistic regression

As shown in **Table 2**, two set of hierarchical logistic regression were performed. In PART 1, models 1~4 evaluated the correlational contributions of all metabolic biomarkers and brain metrics. Interaction and mediation analyses further detected significant factors in the PCOS-depression association. In PART 2, models 5~7 assessed the contributions of these selected factors.

**Table 2:** Logistic regression of the association between PCOS and depression.

Models	Variable	Conditional logistic regression analysis		Bayesian logistic regression analysis	
		OR(95% CI)	p-value	OR (95% CrI)	Pd
PART 1					
Model 1	PCOS	1.784 (1.298~2.452)	<0.001 **	1.795 (1.775~2.332)	100% **
Model 2	PCOS	1.765 (1.276~2.442)	<0.001 **	1.865 (1.794~2.419)	100% **
Model 3	PCOS	1.602 (1.141~2.248)	0.006 **	-	-
Metabolic biomarkers	BMI	1.058 (1.020~1.097)	0.003 **	-	-
	FAI	0.903 (0.818~0.997)	0.044 *	-	-
	SII	1.001 (1.000~1.001)	0.011 *	-	-
	TyG	1.279 (0.882~1.853)	0.194	-	-
Model 4	PCOS	-	-	0.905~1.153 (straddling zero)	Not significant
Regional brain volume	Hippocampus GMVol	-	-	1.131 (0.769~1.665)	73.10%
	Amygdala GMVol	-	-	0.724 (0.450~1.159)	90.80%
	FMC GMVol	-	-	1.011 (0.694~1.453)	51.80%
	FOC GMVol	-	-	0.922 (0.577~1.456)	63.70%
	Accumbens Vol	-	-	1.083 (0.727~1.573)	65.00%
	Thalamus Vol	-	-	1.388 (0.780~2.528)	87.50%
Cortical thickness	RostAntCing Thk	-	-	1.231 (0.262~5.137)	59.70%
	CaudalAntCing Thk	-	-	0.461 (0.156~1.360)	92.60%
	Insula Thk	-	-	0.559 (0.127~2.638)	77.50%
	MedOrbFront Thk	-	-	0.811 (0.192~3.485)	60.80%
	LatOrbFront Thk	-	-	0.745 (0.146~3.981)	64.30%
dMRI metrics	CH FA	-	-	0.881 (0.451~5.653)	56.20%
	CH ICVF	-	-	0.869 (0.490~5.747)	55.30%
	CCG FA	-	-	1.077 (0.519~6.806)	53.20%
	CCG ICVF	-	-	0.753 (0.457~5.437)	61.30%
	UF FA	-	-	0.867 (0.276~5.912)	55.90%
	UF ICVF	-	-	0.931 (0.352~6.302)	52.60%
Task-based fMRI	Amygdala BOLD Effect	-	-	0.937 (0.207~4.172)	53.30%
PART 2					
Model 5	PCOS	-	-	1.777 (1.341~2.394)	100% **
	BMI	-	-	1.271 (1.114~1.438)	99.80% **
	FAI	-	-	0.830 (0.710~0.965)	99.60% **
	SII	-	-	1.258 (1.129~1.401)	100% **
	TyG	-	-	1.111 (0.969~1.267)	93.20%
Model 6	PCOS	-	-	1.171 (0.617~2.399)	66.70%
	FOC GMVol	-	-	0.986 (0.721~1.348)	54.70%
	CaudalAntCing Thk	-	-	0.769 (0.565~1.010)	96.80% †
Model 7	PCOS	-	-	1.002 (0.420~2.329)	50.30%
	BMI	-	-	0.912 (0.620~1.409)	68.80%
	FAI	-	-	1.177 (0.775~1.738)	79.60%
	SII	-	-	1.245 (0.917~1.703)	93.30%
	TyG	-	-	1.138 (0.756~1.687)	74.50%
	FOC GMVol	-	-	0.953 (0.665~1.353)	60.80%
	CaudalAntCing Thk	-	-	0.667 (0.481~0.937)	99.20% *

PART 1: Preliminarily evaluated the contributions of metabolic biomarkers and brain MRI metrics.

PATR 2: Performed after interactive and mediating analyses. The covariates were specified as chosen metabolic biomarkers and brain MRI metrics.

Model 1: Crude model.

Model 2: Adjusted for basic covariates including age at recruitment, ethnicity, education level, physical activity, smoking status, alcohol consumption and menopause status.

Model 3: Adjusted for Model 2 covariates + metabolic biomarkers in conditional logistic regression analysis.

Model 4: Adjusted for Model 2 covariates + 18 brain MRI metrics (one brain MRI metric at a time) in Bayesian logistic regression analysis.

Model 5: Adjusted for Model 2 covariates + metabolic biomarkers in Bayesian logistic regression analysis.

Model 6: Adjusted for Model 2 covariates + 2 brain MRI metrics (FOC GMVol and CaudalAntCing Thk) in Bayesian logistic regression analysis.

Model 7: Adjusted for Model 2 covariates + metabolic biomarkers + brain MRI metrics (FOC GMVol and CaudalAntCing Thk) in Bayesian logistic regression analysis.

Data were presented as OR with 95% CI/CrI. *p-value<0.05, **p-value<0.01 or *Pd >97.5%,**Pd >99.5% were considered statistically significant. A marginal trend was defined as † Pd closed to 97.5%, indicating a tendency without statistical robustness.

PART 1: PCOS was significantly associated with depression in Model 1(OR = 1.784, 95% CI: 1.298~2.452, p< 0.001). This association remained significant after basic covariate-adjustment in Model 2(OR = 1.765, 95% CI: 1.276~2.442, p< 0.001). Further metabolic adjustment in Model 3 slightly attenuated but did not eliminate the association(OR = 1.602, 95% CI: 1.141~2.248, p =0.006). However, when brain metrics were included in Model 4, the association was no longer significant. These results indicated that metabolic biomarkers only partially accounted for the PCOS-depression association, whereas brain metrics played more critical roles in this association. Additionally, BMI, FAI and SII were significantly associated with depression in Model 3, whereas TyG showed no significance, suggesting that BMI, FAI and SII displayed independent links to depression beyond PCOS itself.

PART 2: Results from Bayesian logistic models 1, 2, and 5 corroborated those from conditional logistic models 1-3. In Model 6, CaudalAntCing Thk showed only a marginal trend, whereas in Model 7 it became significant(OR = 0.667, 95% HDI = 0.481~0.937, Pd = 99.20%), with PCOS and all metabolic biomarkers being non-significant, suggesting that CaudalAntCing Thk played a prominent role in depression.

### Interactive effects of brain metrics and mediating effects of metabolic biomarkers

As shown in **Table 3**; **Supplementary Table 1**, neither multiplicative interaction nor additive interaction was observed between PCOS and metabolic biomarkers. However, multiplicative interactions were detected between PCOS and two brain metrics: FOC GMVol(β = 0.182, 95% HDI: 0.080~0.950, ROPE = 0.03%) and CaudalAntCing Thk(β = 0.121, 95% HDI: 0.016~0.950, ROPE = 0.36%), indicating that PCOS-depression association was significantly moderated by FOC GMVol and CaudalAntCing Thk, but not by metabolic biomarkers.

**Table 3:** Interactive effects of brain metrics, mediating effects of metabolic biomarkers and interrelationship among metabolic biomarkers.

Variable	β (95% HDI) or β/Bootstrap β (95% CI)	ROPE or p-value	Total Effect (%)
Interaction analyses			
PCOS	0.053 (-0.050~0.961)	0.84%	–
FOC GMVol	-0.047 (-0.093~0.953)	1.00%	–
PCOS×FOC GMVol	0.182 (0.080~0.950) *	0.03%	–
PCOS	0.044 (-0.060~0.954)	0.88%	–
CaudalAntCing Thk	-0.069 (-0.113~0.962)	0.93%	–
PCOS×CaudalAntCing Thk	0.121 (0.016~0.950) *	0.36%	–
Mediation analyses: PART 1			
PCOS→BMI	1.642 (1.042~2.263)	<0.001 **	–
BMI→depression	0.007 (0.003~0.011)	<0.001 **	–
PCOS→BMI→depression	0.012 (0.005~ 0.020) *	–	13.00%
PCOS→FAI	0.401 (0.154~0.682)	0.003 **	–
FAI→depression	-0.011 (-0.019~ -0.003)	0.008 **	–
PCOS→FAI→depression	-0.004 (-0.010~ -0.001) *	–	-6.30%
PCOS→SII	20.369 (-9.878~50.562)	0.198	–
SII→depression	0.0001 (0.0001~0.0002)	<0.001 **	–
PCOS→SII→depression	0.002 (-0.001~0.007)	–	–
PCOS→TyG	0.011 (-0.046~0.065)	0.694	–
TyG→depression	0.034 (0.003~0.066)	0.032 **	–
PCOS→TyG→depression	0.0004 (-0.0017~0.0027)	–	–
Mediation analyses: PART 2			
BMI→FAI	0.118 (0.099~0.136)	<0.001 **	–
FAI→depression	0.987 (0.978~0.996)	0.006 **	–
BMI→FAI→depression	0.998 (0.997~1.000) *	–	-27.20%
FAI→BMI	0.770 (0.639~0.879)	<0.001 **	–
BMI→depression	1.007 (1.004~1.011)	<0.001 **	–
FAI→BMI→depression	1.005 (1.003~1.009) *	–	-73.20%
BMI→TyG	0.039 (0.034~0.043)	<0.001 **	–
TyG→depression	1.039 (1.007~1.075)	0.018 *	–
BMI→TyG→depression	1.001 (1.000~1.003) *	–	17.80%
TyG→BMI	3.103 (2.762~3.515)	<0.001 **	–
BMI→depression	1.007 (1.003~1.011)	<0.001 **	–
TyG→BMI→depression	1.021 (1.011~1.034) *	–	35.70%
FAI→TyG	0.011 (-0.003~0.023)	0.073	–
TyG→depression	1.039 (1.008~1.073)	0.017 *	–
FAI→TyG→depression	1.000 (1.000~1.001)	–	–
TyG→FAI	0.134 (-0.032~0.275)	0.073	–
FAI→depression	0.987 (0.978~0.996)	0.005 **	–
TyG→FAI→depression	0.998 (0.997~0.999)	–	–

Interactive Analyses: only FOC GMVol and CaudalAntCing Thk showed interactive effects. Data were presented as β coefficients with 95% HDIs. Statistical significance was set as * 95% HDI excluded zero. ROPE< 2.5% suggested practical significance.

Mediation Analyses: only metabolic biomarkers showed mediating effects.

PART 1: the mediating effects of metabolic biomarkers in PCOS-depression association.

PART 2: the interrelationships among various metabolic biomarkers.

Data were presented as β coefficients (direct effect) or Bootstrap β (indirect effect) with 95% CI. Statistical significance was set at *p< 0.05, **p< 0.01, or *95% CI not containing zero.

Shown in **Table 3**(Mediation Analyses PART1), BMI and FAI exhibited significant mediating effects. Specifically, PCOS was positively associated with BMI, and BMI with depression, resulting in a significant indirect effect of PCOS on depression via BMI(β = 0.012, 95% CI: 0.005~0.020), accounting for 13% of the total effect. As to FAI, PCOS was positively associated with FAI, while FAI inversely with depression, resulting in a negative indirect effect(β = −0.004, 95% CI: −0.010 to −0.001), accounting for −6.30% of the total effect. By contrast, SII or TyG was directly associated with depression, however, the paths from PCOS to SII or TyG were non-significant, indicating no mediating roles of SII and TyG. For brain metrics, as shown in **Supplementary Table 2**, neither direct effect nor indirect effect showed significance.

### Relative weight and PCA

As shown in **Supplementary Table 3**, PCOS status exerted negligible predictive power for depression after all-adjustment, with a relative weight of 0.28%. Metabolic biomarkers collectively weighted 12.60%, among which SII occupied 9.70%, followed by TyG 2.33%, FAI 2.28% and BMI 0.29%. Brain metrics accounted for 20.33%, with CaudalAntCing Thk as the major component(19.49%) whereas FOC GMVol showed a minimal contribution(0.84%).The full model achieved a 10.89% weighted R², explaining more than 10% of the variance in depression, which was a meaningful result in the psychiatric field, given the multifactorial nature of depression.

Three independent components were extracted in PCA(**Supplementary Tables 3**, **4**): PC1(metabolic factors) loaded by BMI, FAI, and TyG explained 26.80% of the total variance. PC2(brain metrics) characterized by FOC GMVol and CaudalAntCing Thk accounted for 19.20%. PC3 dominated by inflammatory SII explained 17.40%.

### Interrelationships among linear dose-response metabolic biomarkers

All metabolic factors exhibited dose-dependent linear patterns in PCOS-depression association with no evidence of threshold(**Supplementary Table 5**). Based on PCA result, SII was excluded in the following analyses. After adjusting for FOC GMVol and CaudalAntCing Thk, the indirect effects of PCOS→BMI/FAI→depression became non-significant(**Supplementary Table 6**), indicating that the mediating effects of BMI and FAI could be overshadowed once these brain metrics were accounted for.

Shown in **Table 3** (Mediation Analyses PART 2), the indirect effect of BMI→FAI→depression was significant with a mediated proportion of −27.20%. For the reversed FAI →BMI→depression, the indirect effect was also significant with a mediated proportion of −73.20%. −27.20% vs. −73.20% indicated stronger magnitude of BMI relative to FAI. For BMI⇋TyG→depression, both of the indirect effect was significant with the mediated proportion as 17.80% and 35.70% respectively. As the PCOS→TyG path was non-significant, TyG exerted its effect on depression via BMI, rather than acting as a mediator for PCOS. In contrast, the bidirectional FAI⇋TyG→depression was non-significant, suggesting no direct interrelation between FAI and TyG. Thus, BMI was identified as the central hub of the metabolic network.

### Threshold-dependent linear patterns of FOC GMVol and CaudalAntCing Thk independent of metabolic factors

Shown in **Table 4** PART1, FOC GMVol and CaudalAntCing Thk exhibited linear associations with depression, with no evidence of nonlinearity, and these linear associations were significantly modified by PCOS status(PCOS×FOC GMVol: p<0.001; PCOS×CaudalAntCing Thk: p=0.020). Threshold intervals for the two-brain metrics were observed within the physiological range as −0.66~0.21 (p<0.05) and −0.68~0.17 (p<0.05) respectively, representing protective ranges in PCOS. Values below or above these intervals were associated with elevated depression risk.

**Table 4:** Linear and stratified analyses of FOC GMVol and CaudalAntCing Thk in relation to depression.

PART 1 threshold-dependent linear patterns of FOC GMVol and CaudalAntCing Thk				
Variable	PCOS		non-PCOS	
	β (95% CI)	p-value	β (95% CI)	p-value
FOC GMVol				
Nonlinearity test				
FOC GMVol	−0.061 (−0.133~ 0.011)	0.099	−0.061 (−0.133~ 0.011)	0.098
FOC GMVol²	–	–	-0.080 (-0.185~ 0.025)	0.136
PCOS×FOC GMVol²	0.088 (−0.137~ 0.313)	0.444	–	–
Linear model				
PCOS	0.048 (−0.050~ 0.146)	0.338	–	–
FOC GMVol	−0.071 (−0.142~ 0.000)	0.051	−0.070 (−0.141~ 0.000)	0.052
PCOS×FOC GMVol	0.280 (0.124~ 0.437)	<0.001 **	–	–
Johnson-Neyman threshold				
Threshold Interval (z-score)	[-0.66, 0.21]	<0.05 *	–	–
CaudalAntCing thk				
Nonlinearity test				
CaudalAntCing Thk	−0.215 (−0.399~ −0.031)	0.022 *	−0.212 (−0.393~ −0.031)	0.022 *
CaudalAntCing Thk²	–	–	0.183 (−0.230~ 0.596)	0.384
PCOS×CaudalAntCing Thk²	−0.922 (−2.140~ 0.297)	0.138	–	–
Linear model				
PCOS	0.036 (−0.063~ 0.134)	0.475	–	–
CaudalAntCing Thk	−0.242 (−0.416~ −0.069)	0.006 **	−0.239 (−0.409~ −0.068)	0.006 **
PCOS×CaudalAntCing Thk	0.490 (0.078~ 0.902)	0.020 *	–	–
Johnson-Neyman threshold				
Threshold Interval (z-score)	[-0.68, 0.17]	<0.05 *	–	–
PART 2 FOC GMVol and CaudalAntCing Thk stratified by PCOS				
Variable	PCOS		non-PCOS	
	OR (95% CrI/CI)	Pd (%)/p-value	OR (95% CrI/CI)	Pd (%)/p-value
FOC GMVol				
Bayesian regression				
Before Metabolic Adjustment	3.434 (1.316~9.603)	99.6 **	0.575 (0.319~0.982)	97.9 *
After Metabolic Adjustment	2.502 (0.788-8.245)	93.7	0.600 (0.314-1.098)	95.4
Stratified logistic regression				
Before Metabolic Adjustment	4.601(1.245~23.234)	0.036 **	0.550 (0.287~1.011)	0.061 †
After Metabolic Adjustment	4.678 (0.714 ~ 77.473)	0.165	0.569 (0.281 ~ 1.103)	0.105
Simple slope				
Before Metabolic Adjustment	1.489 (0.278 ~ 2.699) *	–	-0.588 (-1.204 ~ 0.029)	–
After Metabolic Adjustment	1.250 (-0.112 ~ 2.612)	–	-0.503 (-1.160 ~ 0.154)	–
CaudalAntCing thk				
Bayesian regression				
Before Metabolic Adjustment	1.741 (0.323~9.012)	73.8	0.325 (0.103~1.009)	97.4 †
After Metabolic Adjustment	1.337 (0.214-8.060)	62	0.271 (0.084-0.925)	98.2 *
Stratified logistic regression				
Before Metabolic Adjustment	17.633(0.429~1569.768)	0.159	0.223 (0.053~0.896)	0.036 **
After Metabolic Adjustment	7.454 (0.009 ~ 20565.52)	0.565	0.156 (0.031 ~ 0.730)	0.020 *
Simple slope				
Before Metabolic Adjustment	2.214 (-0.872 ~ 5.300)	–	-1.619 (-3.002 ~ -0.236) *	–
After Metabolic Adjustment	2.402 (-1.661 ~ 6.466)	–	-2.033 (-3.572 ~ -0.494) *	–
PART 3 FOC GMVol and CaudalAntCing thk Stratified by metabolic biomarkers				
Variable	FOC GMVol		CaudalAntCing Thk	
	Interaction term β (95% CI)	P-value	Interaction term β (95% CI)	p-value
BMI Subgroup				
Normal (<25)	0.200 (0.035 ~ 0.365)	0.019 *	0.082 (-0.140 ~ 0.305)	0.47
Overweight (25-29.9)	0.089 (-0.143 ~ 0.321)	0.457	0.120 (-0.130 ~ 0.371)	0.35
Obesity (≥30)	0.113 (-0.420 ~ 0.647)	0.683	-0.119 (-0.521 ~ 0.284)	0.572
FAI subgroup				
Low FAI (≤ median)	-0.017 (-0.186 ~ 0.151)	0.843	0.141 (-0.017 ~ 0.299)	0.084
High FAI (> median)	0.252 (0.071 ~ 0.433)	0.008 **	0.147 (-0.085 ~ 0.379)	0.216
SII subgroup				
Low inflammation (SII< 500)	0.366 (-0.077 ~ 0.808)	0.109	0.051 (-0.177 ~ 0.278)	0.663
High inflammation (SII ≥ 500)	0.104 (-0.042 ~ 0.250)	0.166	0.176 (-0.004 ~ 0.356)	0.058
TyG subgroup				
Non-insulin resistance (TyG< 8.5)	0.161 (-0.001 ~ 0.323)	0.053	0.069 (-0.094 ~ 0.233)	0.407
Insulin resistance (TyG ≥ 8.5)	0.174 (-0.047 ~ 0.394)	0.128	0.241 (-0.010 ~ 0.491)	0.064
Normal-BMI subgroup				
PCOS×FOC GMVol (total)	0.297 (0.101 ~ 0.493)	0.004 **	–	–
Low FAI (≤ median)				
PCOS×FOC GMVol	0.053 (-0.108 ~ 0.213)	0.521	–	–
High FAI (> median)				
PCOS×FOC GMVol	0.330 (0.029 ~ 0.630)	0.036 *	–	–
PCOS×FOC GMVol×FAI	0.288 (0.039 ~ 0.537)	0.028 *	–	–

PART1: Nonlinearity was assessed by including a quadratic term and its interaction with PCOS; the quadratic term was non-significant, supporting linearity. Johnson–Neyman intervals were subsequently identified within the linear model.

PRAT 2: PCOS-stratified analyses were performed using three approaches: (1) Bayesian regression, (2) stratified logistic regression, and (3) simple slope analysis to interpret interaction effects within each group.

PART 3: Metabolic-stratified analyses were performed and followed by FAI subgrouping among normal-BMI participants for three-way interaction testing.

Data were presented as β/OR, 95% CI/CrI, and p-value/Pd (%).* p-value<0.05, Pd(%) >97.5, or 95% CI not containing 0; ** p-value<0.01, Pd(%) >99.5 were defined as statistically significant. A marginal trend was defined as † p-value closed to 0.05 and Pd(%) closed to 97.5 in PART 2.

In non-PCOS, the two-brain metrics exhibited negative linear association with depression but no threshold was detected. Moreover, no differences were observed in FOC GMVol or CaudalAntCing Thk between PCOS and non-PCOS either inside or outside the threshold (**Supplementary Figure 2**), suggesting that the threshold-dependent pattern was not related to morphological differences.

The interaction of FOC GMVol and CaudalAntCing Thk remained consistent before and after metabolic adjustment(**Supplementary Table 7**), suggesting that the effects of the brain metrics were independent of metabolic status.

### FOC GMVol and CaudalAntCingThk Stratified by PCOS and by metabolic biomarkers

As shown in **Table 4** PART2, before metabolic adjustment, FOC GMVol showed a subgroup-specific dual effect: it was positively associated with depression in PCOS(Bayesian regression: OR = 3.434, 95% CrI: 1.316~9.603, Pd = 99.6%; stratified logistic regression: OR = 4.601, 95% CI: 1.245~23.234, p = 0.036) but inversely associated with depression in non-PCOS(Bayesian regression: OR = 0.575, 95% CrI: 0.319~0.982, Pd = 97.9%; stratified logistic regression: OR = 0.550, 95% CI: 0.287~1.011, p = 0.061). For CaudalAntCing Thk, a subgroup-specific protective effect was revealed: CaudalAntCing Thk showed no association with depression in PCOS but was inversely associated with depression in non-PCOS(Bayesian regression: OR = 0.325, 95% CrI: 0.103~1.009, Pd =97.4%; stratified logistic regression: OR = 0.223, 95% CI: 0.053~0.896, p = 0.036). Simple slope analyses confirmed these patterns in both FOC GMVol and CaudalAntCingThk.

After metabolic adjustment, the protective effect of CaudalAntCingThk in non-PCOS remained significant(Bayesian regression: OR = 0.271, 95% CrI: 0.084~ 0.92, Pd =98.2%; stratified logistic regression: OR = 0.156, 95% CI: 0.031 ~ 0.730, p = 0.020), whereas the dual-effect of FOC GMVol lost significance, indicating the metabolic-independent protective role of CaudalAntCingThk and the metabolic-dependent dual-effect of FOC GMVol.

Shown in **Table 4** PART3, interactive effects were observed in normal BMI subgroup(β = 0.200, 95% CI: 0.035~0.365, p = 0.019) and high FAI subgroup(β = 0.252, 95% CI: 0.071~0.433, p = 0.008) in the metabolic-dependent FOC GMVol. Then, FAI was further stratified within the normal-BMI subgroup. A PCOS×FOC GMVol interaction and even a PCOS×FOC GMVol×FAI three-way interaction was observed(β = 0.288, 95% CI: 0.039~0.537, p = 0.028) only in the high FAI subgroup with normal BMI.

### FOC GMVol-CaudalAntCing Thk association stratified by metabolic biomarkers and by PCOS

No mediating or interactive effects were observed between the two-brain metrics to depression. However, a positive bidirectional association of FOC GMVol-CaudalAntCing Thk was detected before or after metabolic adjustment (**Supplementary Table 8**, **9**).

Stratified by metabolic biomarkers (**Table 5**), the FOC GMVol-CaudalAntCing Thk association was significant in overweight/obesity subgroup(β=0.237, 95% CI: 0.049~0.424, p=0.015), marginally significant in high FAI subgroup and high SII subgroup, but non-significant in TyG subgroups. Given that SII was excluded from the metabolic PC1, BMI and FAI were further stratified by PCOS. The FOC GMVol-CaudalAntCing Thk association was retained in non-PCOS with overweight/obesity(β=0.249, 95% CI: 0.027~0.471, p=0.031) or with high FAI(marginally significant) but absent in PCOS. In even further stratification, non-PCOS participants were cross-classified by FAI(low, high) and BMI(normal, overweight/obesity). The FOC GMVol-CaudalAntCing Thk association was only marginally significant in high FAI plus overweight/obesity(β= 0.205, 95% CI: -0.005~0.414, p=0.062) subgroup, indicating that the association tended to occur under the concurrent condition of both high FAI and high BMI.

**Table 5:** FOC GMVol-CaudalAntCing Thk association stratified by metabolic biomarkers and by PCOS.

Subgroups	Mediator and path	β (95% CI)	p-value
Normal BMI	FOC GMVol → CaudalAntCing Thk	0.088 (-0.095~0.270)	0.350
Overweight/Obesity	FOC GMVol → CaudalAntCing Thk	0.237 (0.049~0.424)	0.015 *
Low FAI	FOC GMVol → CaudalAntCing Thk	0.070 (-0.146~0.286)	0.527
High FAI	FOC GMVol → CaudalAntCing Thk	0.163 (0.000~0.325)	0.053 †
Low inflammation	FOC GMVol → CaudalAntCing Thk	0.093 (-0.138~0.323)	0.432
High inflammation	FOC GMVol → CaudalAntCing Thk	0.150 (-0.010~0.310)	0.069 †
Non-insulin resistance	FOC GMVol → CaudalAntCing Thk	0.098 (-0.065–0.260)	0.240
Insulin resistance	FOC GMVol → CaudalAntCing Thk	0.205 (-0.034–0.445)	0.098
Overweight/Obesity + PCOS	FOC GMVol → CaudalAntCing Thk	0.263 (-0.150~0.676)	0.210
	BMI → FOC GMVol	0.132 (-0.300~0.564)	0.546
	BMI → CaudalAntCing Thk	-0.074 (-0.392~0.244)	0.641
	FAI → FOC GMVol	-0.141 (-0.464~0.182)	0.398
	FAI → CaudalAntCing Thk	-0.236 (-0.496~0.024)	0.069 †
Overweight/Obesity + non-PCOS	FOC GMVol → CaudalAntCing Thk	0.249 (0.027~0.471)	0.031 *
	BMI → FOC GMVol	0.032 (-0.306~0.370)	0.843
	BMI → CaudalAntCing Thk	-0.090 (-0.386~0.206)	0.557
	FAI → FOC GMVol	-0.138 (-0.347~0.071)	0.195
	FAI → CaudalAntCing Thk	0.059 (-0.151~0.269)	0.562
High FAI + PCOS	FOC GMVol → CaudalAntCing Thk	0.219 (-0.202~0.640)	0.304
	BMI → FOC GMVol	-0.084 (-0.586~0.418)	0.752
	BMI → CaudalAntCing Thk	-0.093 (-0.492~0.306)	0.640
	FAI → FOC GMVol	-0.097 (-0.511~0.317)	0.646
	FAI → CaudalAntCing Thk	-0.098 (-0.404~0.208)	0.534
High FAI + non-PCOS	FOC GMVol → CaudalAntCing Thk	0.187 (0.001~0.373)	0.051 †
	BMI → FOC GMVol	-0.039 (-0.326~0.248)	0.777
	BMI → CaudalAntCing Thk	0.062 (-0.164~0.288)	0.580
	FAI → FOC GMVol	-0.289 (-0.577~ -0.001)	0.050 †
	FAI → CaudalAntCing Thk	0.098 (-0.146~0.342)	0.426
Non-PCOS + Low FAI + Normal BMI	FOC GMVol → CaudalAntCing Thk	0.061 (-0.226~0.349)	0.677
Non-PCOS + Low FAI + Overweight/Obesity	FOC GMVol → CaudalAntCing Thk	0.191 (-0.298~0.679)	0.451
Non-PCOS + High FAI + Normal BMI	FOC GMVol → CaudalAntCing Thk	0.121 (-0.154~0.395)	0.396
Non-PCOS + High FAI + Overweight/Obesity	FOC GMVol → CaudalAntCing Thk	0.205 (-0.005~0.414)	0.062 †

Data were presented as β with 95% CIs. *A 95% CI not crossing zero and p-value<0.05 was considered statistically significant. A marginally significance was defined as †p-value close to 0.05.

Interestingly, an association of FAI-CaudalAntCing Thk in overweight/obesity PCOS as well as another association of FAI-FOC GMVol in high FAI non-PCOS were observed, suggesting FAI correlated with both FOC GMVol and CaudalAntCing Thk in specific population respectively.

## Discussion

PCOS exerts lifelong impacts from adolescence to post menopause with a persistently elevated risk of depression that substantially impairs quality of life. However, the underlying mechanism linking PCOS to depression remains incompletely elucidated. Previously researches concentrate on the endocrine axes and metabolic risk factors (17). In recent years, growing interest turns to brain structural changes associated with depressive symptoms in patients with PCOS (18–20). In this study, the metabolic and brain structural pathways underlying depression in PCOS were systematically investigated with the key findings described as follows.

### Dominant role of brain metrics and CaudalAntCing Thk as final common executor in PCOS-depression

The caudal anterior cingulate cortex(ACC) is a key node in the cognitive control network and has been implicated in depression pathophysiology, particularly in conflict monitoring, cognitive dyscontrol, and emotion-cognition interaction (21–24). Greater CaudalAntCing Thk has been associated with better improvement in depressive symptoms, suggesting a protective role (10, 25). In PCOS, whole-brain fMRI has shown greater activation in the prefrontal and ventral ACC during emotional processing compared with healthy controls (26), and animal models have further linked DHEA-induced PCOS to depressive-like behaviors with structural changes in the mPFC and ACC (19). The existing evidence support a link between ACC and depression in PCOS, however, no study has specifically examined the caudal subregion of the ACC in the PCOS-depression association.

In this study, PCOS as well as metabolic biomarkers lost association with depression after brain metrics adjustment, suggesting that it was brain structural changes, but not metabolic disturbances, might occupy a prominent position in PCOS-related depression. After adjustment, only CaudalAntCing Thk(caudal ACC) remained significantly associated with depression, indicating it acted as the final common executor underlying PCOS-related depression, while metabolic factors might correlate with depression indirectly through CaudalAntCing Thk. CaudalAntCing Thk also exhibited the highest relative weight(19.49%), far beyond the other factors(PCOS status 0.28; BMI 0.29; FAI 2.28; TyG 2.33; SII 9.70; FOC GMVol 0.84), further supporting its critical role. Moreover, consistent with previous studies (10, 25), the protective effect of CaudalAntCing Thk against depression was confirmed in this study. However, in contrast to CaudalAntCing Thk, RostAntCing Thk(rostral ACC) showed no correlation with depression in this study, highlighting that only the caudal subregion of ACC, not the rostral, exerted protective effects against depression.

### Interaction between PCOS and brain metrics: metabolism-dependent FOC GMVol vs. metabolism-independent CaudalAntCing Thk, and elevated depression risk beyond thresholds

The interactions of FOC GMVol and CaudalAntCing Thk with PCOS(PCOS×FOC GMVol, PCOS×CaudalAntCing Thk) were observed in this study and these interactions maintained significant before and after metabolic adjustment. Both FOC GMVol and CaudalAntCing Thk exhibited interactive effects but not mediating effects on PCOS-related depression, indicating that the roles of brain structures were modulatory rather than mediational. The other brain structures including amygdala, hippocampus, accumbens, thalamus, insula, cingulum cingulate gyrus, cingulum hippocampus and uncinate fasciculus showed no interactive or mediating effect on PCOS-related depression.

The orbitofrontal cortex(OFC; measured as FOC GMVol in this study) is a prefrontal region involved in emotional and reward processing and plays a critical role in depression (27). Depression has been linked to hyperresponsivity in the lateral OFC and hyporesponsivity in the medial OFC (28, 29). In PCOS, elevated OFC activity has been observed in an insulin-sensitivity-dependent manner (30). Thus, although the OFC is implicated in both depression and PCOS, its role in PCOS-related depression remains unexplored.

In this study, the metabolism-dependent dual-effect of FOC GMVol(frontal orbital OFC) was identified: elevating depressive risk in PCOS but protecting against depression in non-PCOS only before metabolic adjustment. Furthermore, a PCOS×FAI×FOC GMVol three-way interaction was detected in normal BMI individuals with high FAI, indicating the highest depression risk in lean PCOS patients with hyperandrogenism. This three-way interaction might be biologically plausible given the expression of androgen receptors (AR) within the OFC, as well as reported association between AR (CAG)n polymorphisms and OFC gray matter volume (31, 32). For CaudalAntCing Thk, a protective effect was observed in non-PCOS, but no effect detected in PCOS, and this pattern persisted independent of metabolic status. Collectively, the metabolism-dependent effects of FOC GMVol versus the metabolism-independent CaudalAntCing Thk illustrated prominent region-specific heterogeneity. Speculatively, as a potential integrative hub, the metabolism-dependent FOC GMVol might perceive metabolic inputs, evaluate emotional valence and reward, and send descending signals toward the metabolism-independent CaudalAntCing Thk for conflict monitoring and behavioral execution, thereby jointly correlating with depression risk in PCOS behavior.

Furthermore, distinct from non-PCOS without threshold, the threshold-dependent linear patterns of FOC GMVol and CaudalAntCing Thk were observed in PCOS-depression association, suggesting that PCOS patients tended to show higher depression risk beyond these threshold intervals. However, the threshold-dependent patterns were not related to morphological differences. Therefore, PCOS might not directly alter brain morphological structure. Instead, it might be linked to shifts in the intrinsic patterns of FOC GMVol and CaudalAntCing Thk, switching from a no-threshold pattern in non-PCOS to a threshold-dependent pattern in PCOS.

Interestingly, the interaction was detected between PCOS and FOC gray matter volume(PCOS×FOC GMVol), but not in MedOrbFront Thk and LatOrbFront Thk(medial or lateral of FOC cortical thickness), suggesting that PCOS-related alterations might preferentially related to the overall FOC rather than its subregions. Or, gray matter volume might represent a more sensitive measure than cortical thickness for detecting this interaction. In addition to OFC, we also examined the medial prefrontal cortex(mPFC), another prefrontal subdivision measured as FMC GMVol. Although multiple human neuroimaging and animal studies suggest that this subregion is involved in the pathogenesis of depression or PCOS (11, 19, 33, 34), FMC GMVol showed no association with PCOS-related depression in this study.

### FOC GMVol-CaudalAntCing Thk association activated by concurrent increases of BMI and FAI in non-PCOS

Neuroimaging, neuropsychological and animal studies have established distributed connections between the OFC and ACC. During decision-making, caudal ACC engages in ambiguous choices while OFC links to risky decisions, reflecting complementary “cool” cognitive and “hot” affective contributions within an OFC-ACC network (35). Moreover, OFC and ACC cooperate through the cognitive control network making them promising MRI-based depression biomarkers (36). Although the roles of OFC-ACC in depression have been established, their association in PCOS-related depression remains unexplored.

In our study, a bidirectional FOC GMVol-CaudalAntCing Thk association exclusively in non-PCOS was observed with no interaction or mediation between the two metrics in relation to depression, suggesting that this structural coupling might be absent in PCOS. Moreover, the association was maintained only by the combination of high BMI and high FAI in non-PCOS, whereas high FAI or high BMI alone was insufficient. These findings suggested that concurrent increases in BMI and FAI might act as a trigger to active this latent structural coupling in non-PCOS.

### Two-tiered protective hypothesis in brain to PCOS-depression

Thus, we proposed a “two-tiered protective hypothesis” in brain including “neural compensatory coupling” and “protective threshold” to elucidate the involvement of brain metrics in PCOS-related depression.

In non-PCOS, metabolic abnormalities(obesity and hyperandrogenism) might correlate with an adaptive neural response, manifested as the unmasked structural coupling of FOC GMVol-CaudalAntCing Thk. This coupling might reflect an endogenous compensatory mechanism to preserve emotion-cognition integration and counteract the depressogenic effects of metabolic stress. Crucially, this compensatory coupling was not present in PCOS, despite comparable levels of metabolic burden. This PCOS-specific difference, possibly related to the dysregulation of hypothalamic-pituitary-ovarian(HPO) axis or hypothalamic-pituitary-adrenal(HPA) axis or inherent vulnerability, might serve as a switch selectively abolished the brain’s adaptive response to metabolic challenge. Consequently, although metabolic stress corresponded to protective neural compensation in non-PCOS, the same metabolic stress failed to elicit this response in PCOS, leaving the brain vulnerable to depression.

Then, among PCOS patients who lost the compensatory FOC GMVol-CaudalAntCing Thk coupling, the threshold-dependent protective mechanism might initiate: individuals with FOC GMVol and CaudalAntCing Thk inside the threshold ranges could still be buffered from depression susceptibility. In contrast, PCOS patients whose FOC GMVol and CaudalAntCing Thk values fell outside the threshold ranges exhibited higher depressive vulnerability.

### Linear mediating effects of BMI and FAI in PCOS-depression

Elevated depression risk in PCOS is reported to closely link to hyperandrogenism, insulin resistance, obesity and chronic low-grade inflammation (3, 7, 17, 37–41). In this study, BMI and SII emerged as risk factors while FAI as protective factor for depression in a dose-dependent linear patterns. Both BMI and FAI showed mediation in the PCOS-depression association, with BMI’s positive mediating effect outweighing the negative effect of FAI, although these mediation effects could be overshadowed by brain metrics. SII was detected as an independent risk factor to depression irrelevant to PCOS.

Although obesity and depression are positively and bidirectionally associated (13, 42, 43), elevated BMI might partly but not fully explain the increased depression risk in PCOS, as lean PCOS women also show higher depression prevalence than lean controls (44–46). Unlike obesity whose detrimental effect on depression is well established, the role of androgen in depression remains controversial. Most studies report no significant association between physiological androgen levels and female depression (5, 47–49). However, in PCOS, a positive association between FAI and depression mediated by BMI is reported (50), and both androgen-lowering and weight loss interventions have been shown to alleviate depressive symptoms in overweight/obese PCOS women (51–53). Our findings corroborated the harmful effect of BMI on depression in PCOS, consistent with previous reports. Notably, we observed a weak but protective peripheral mediating effect of FAI, which seemed divergent from prior evidence. However, this subtle beneficial peripheral effect of FAI could be outweighed by the stronger BMI-driven detrimental mediation, providing a plausible explanation for the previously observed positive FAI-depression association (50).

### Metabolic network in PCOS-depression: BMI as a hub linking antagonistic FAI and auxiliary TyG

In this study, a metabolic network was observed: detrimental BMI outweighed protective FAI in the BMI⇋FAI loop while TyG reinforced BMI in the BMI⇋TyG loop, with no interrelation between FAI and TyG. Therefore, TyG showed no mediation in the PCOS-depression association but exerted auxiliary influence to BMI, and BMI was identified as a hub linking FAI and TyG.

Meanwhile, the rank of relative weights as SII > TyG > FAI > BMI > PCOS status indicated that without the influence of PCOS, inflammation, insulin resistance and hyperandrogenism were stronger correlates of depression than BMI. However, in PCOS, BMI served as a dominant hub mediator linking PCOS to depression. Thus, the harmful effect of obesity on depression might be magnified in PCOS.

### Distinct roles of BMI and FAI at central versus peripheral level: FAI played more critical roles than BMI in PCOS-depression

At central level, high FAI combined with high BMI to activate FOC GMVol-CaudalAntCing Thk coupling in non-PCOS. Meanwhile, FAI inhibited FOC GMVol in the same group. In PCOS, FAI involved in a three-way interaction of PCOS×FAI×FOC GMVol under normal-BMI and high FAI condition. Concurrently, FAI inhibited CaudalAntCing Thk in overweight/obesity PCOS. At peripheral level, FAI exhibited a protective mediating effect which could be antagonized by BMI. As to BMI, it acted as a detrimental mediator at peripheral level, and appeared to facilitate the effects of FAI at central level. These findings highlighted that it was FAI rather than BMI, although some FAI’s effects BMI-dependent, that played critical roles in PCOS-depression association. Thus, FAI might be considered as a vital versatile regulator linking metabolism and brain structure with its direction and strength jointly influenced by BMI, PCOS status, and brain metrics. Specifically, FAI exerted pronounced harmful actions at central level and a faint protective peripheral effect in PCOS, coupled with its BMI-dependent trait.

Given these findings, a synergistic therapeutic strategy of androgen suppression plus weight management was recommended. In obese women with PCOS, weight loss conferred both central and peripheral benefits should be recommended. However, caution was warranted when weight loss approached the normal-BMI range. In normal-BMI PCOS with hyperandrogenism, the three-way interaction of PCOS×FAI×FOC GMVol indicated an even higher risk of depression, although whether the underlying mechanisms of depression in obesity versus normal-BMI PCOS differ remained to be determined.

## Conclusions, limitations, and future directions

This study provided a new perspective to the PCOS-depression framework, shown in **Figure 1**, highlighting that in this framework brain structures exerted dominant roles with CaudalAntCing Thk as the final common executor. BMI and FAI exhibited mediating effects in PCOS-depression association, while SII acted as an independent risk factor to depression irrelevant to PCOS. A two-tiered brain protective hypothesis was proposed against depression: neural compensation reflected the protective FOC GMVol-CaudalAntCing Thk coupling in non-PCOS, and protective threshold patterns of both FOC GMVol and CaudalAntCing Thk in PCOS, with FAI playing pivotal and distinct roles at peripheral and central levels across the entire pathways.

**Figure 1:**
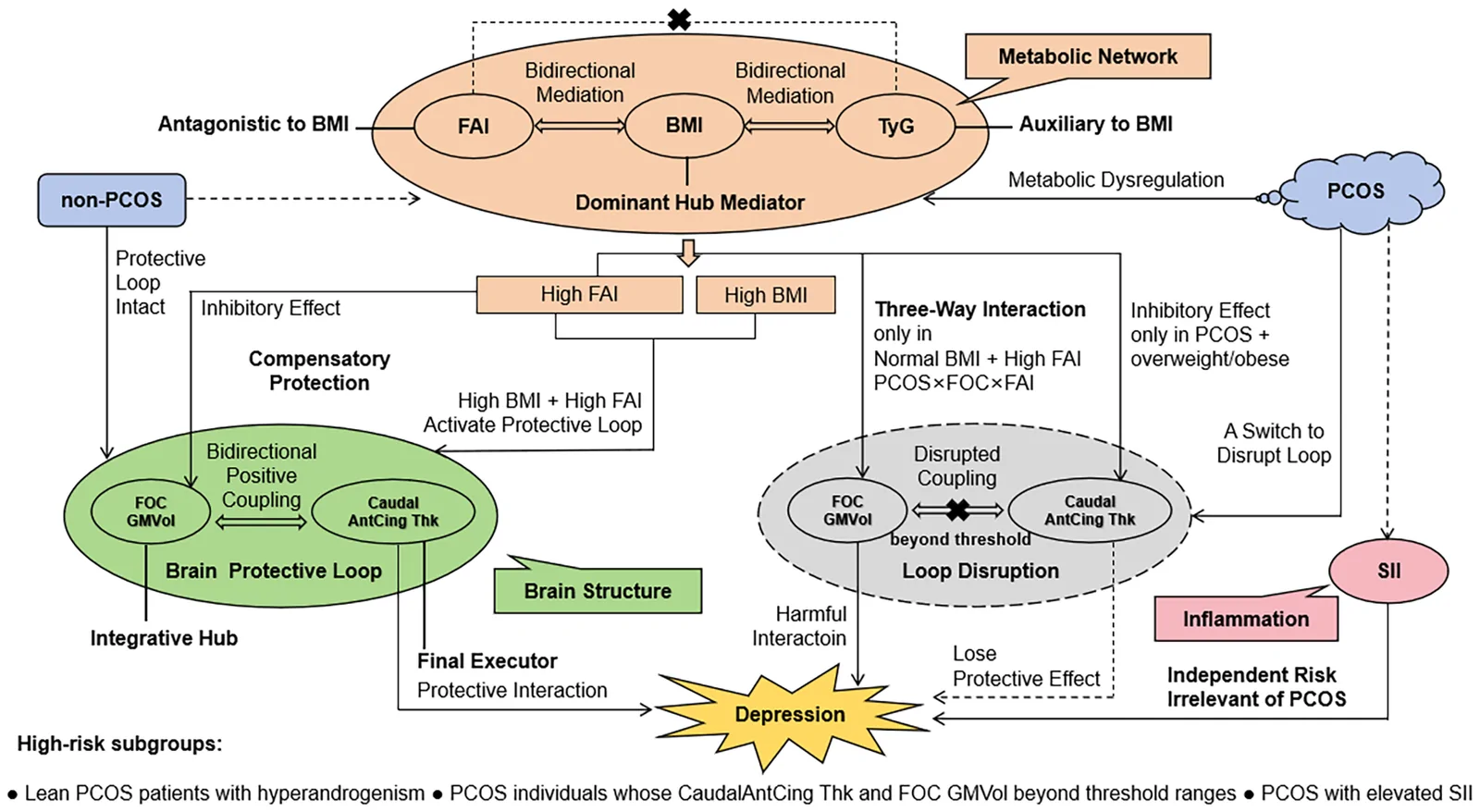
Integrative framework of neuro-metabolic correlates in PCOS-depression.

However, this study had several limitations. First, the cross-sectional design precluded causal inference in this study due to sample size limitations. Second, brain structural metrics were based on MRI and did not incorporate functional imaging or neurobiochemical measures. Metabolic indices were limited to a subset of biomarkers and did not cover adipokines, the gut-brain axis, or other relevant factors. Third, potential selection bias in the study population from UK Biobank might limit the generalizability of the present findings. Fourth, only sensitivity analyses for metformin use were provided in **Supplementary Table 10**, whereas confounding effects of other medications required further verification. Future studies should employ longitudinal designs and integrate functional imaging with metabolomics to further clarify the neuro-metabolic mechanisms.

At the central structural level:

In non-PCOS(left), when high BMI and high FAI co-occurred, the FOC GMVol-CaudalAntCing Thk compensatory loop displayed a protective effect against depression, with CaudalAntCing Thk serving as the final executor and FOC GMVol as a integrative hub that perceived metabolic inputs and projected descending signals to CaudalAntCing Thk. Meanwhile, FAI showed an inverse correlation with FOC GMVol under these circumstances.

In PCOS(right), PCOS as a switch correlated with the absence of the FOC GMVol-CaudalAntCing Thk loop. FAI was linked to elevated depression vulnerability via the PCOS×FOC×FAI three-way interaction in normal BMI with high FAI. Meanwhile, FAI showed an inverse correlation with the protective CaudalAntCing Thk in overweight/obesity PCOS. Ultimately, PCOS patients whose FOC GMVol and CaudalAntCing Thk values fell outside the threshold ranges exhibited higher depressive vulnerability.

At the peripheral level (top):

BMI and FAI exhibited mediating effects with BMI serving as a hub linking antagonistic FAI and auxiliary TyG, although these mediation effects could be overshadowed by brain metrics. SII was detected as an independent risk factor to depression irrelevant to PCOS.

High-risk subgroups were highlighted as below (bottom).

## Data Availability

The raw data supporting the conclusions of this article will be made available by the authors, without undue reservation.

## Glossary

PCOS: polycystic ovary syndrome
PCA: principal component analysis
MRI: magnetic resonance imaging
FOC GMVol: volume of grey matter in frontal orbital cortex
CaudalAntCing Thk: mean thickness of caudalanteriorcingulate
FAI: free androgen index
BMI: body mass index
TyG: triglyceride-glucose index
SII: systemic immune-inflammation index
SHBG: sex hormone-binding globulin
dMRI: diffusion MRI
FA: fractional anisotropy
ICVF: intravoxel incoherent motion
fMRI: functional MRI
IQR: interquartile range
SEM: structural equation modeling
LMG: Lindeman, Merenda, and Gold
GAMs: generalized additive models
edf: effective degrees of freedom
OR: odds ratios
CI: confidence interval
CrI: credible interval
HDIs: highest density intervals
Pd: probability of direction
Hippocampus GMVol: volume of grey matter in hippocampus
Amygdala GMVol: volume of grey matter in amygdala
FMC GMVol: volume of grey matter in frontal medial cortex
mPFC: frontal medial cortex corresponded to medial prefrontal cortex
FOC GMVol: volume of grey matter in frontal orbital cortex
OFC: frontal orbital cortex corresponded to orbitofrontal cortex
Accumbens Vol: volume of accumbens
Thalamus Vol: volume of thalamus
RostAntCing Thk: mean thickness of rostralanteriorcingulate
rostral ACC: rostralanteriorcingulate corresponded to rostral anterior cingulate cortex
CaudalAntCing Thk: mean thickness of caudalanteriorcingulate
caudal ACC: caudalanteriorcingulate corresponded to caudal anterior cingulate cortex
Insula Thk: mean thickness of insula
MedOrbFront Thk: mean thickness of medialorbitofrontal
LatOrbFront Thk: mean thickness of lateralorbitofrontal
CH FA: mean FA in cingulum hippocampus on FA skeleton
CH ICVF: mean ICVF in cingulum hippocampus on FA skeleton
CCG FA: mean FA in cingulum cingulate gyrus on FA skeleton
CCG ICVF: mean ICVF in cingulum cingulate gyrus on FA skeleton
UF FA: mean FA in uncinate fasciculus on FA skeleton
UF ICVF: mean ICVF in uncinate fasciculus on FA skeleton
Amygdala BOLD Effect: 90th percentile of blood oxygen level-dependent effect (in group-defined amygdala activation mask) for faces-shapes contrast
ACC: caudal anterior cingulate cortex
OFC: orbitofrontal cortex
AR: androgen receptors
mPFC: medial prefrontal cortex
HPO axis: hypothalamic-pituitary-ovarian axis
HPA axis: hypothalamic-pituitary-adrenal axis
