# Supplementary material for: From metabolic network to brain loop: unraveling the neuro-metabolic correlates in PCOS-related depression
Source: Front Endocrinol (Lausanne). 2026 Sep 21;17:1925127. doi: 10.3389/fendo.2026.1925127 (PMC13635201; doi:10.3389/fendo.2026.1925127)
Supplement: Supplementary file 1 [file Table1.docx]

| Supplymentary Table 1-1. The Additive Interaction Effects of Metabolic Biomarkers in PCOS-Related Depression | | |
| --- | --- | --- |
| Variable | Additive interaction | |
|  | RERI（95% CI） | p-value |
| PCOS × overweight | -1.223 (-4.45 - 1.088) | 0.205 |
| PCOS × obese | 1.988 (-2.835 - 10.256) | 0.888 |
|  |  |  |
| PCOS × FAI (> 5) | -1.635 (-3.607 - 0.151) | 0.121 |
|  |  |  |
| PCOS × SII (≥500) | 0.162 (-3.337 - 1.508) | 0.898 |
|  |  |  |
| PCOS × TyG (≥8.5) | -0.421 (-2.393 - 1.612) | 0.411 |

| Supplymentary Table 1-2. The Detailed Interactive Effects of Brain MRI Metrics in PCOS-Related Depression | | |
| --- | --- | --- |
|  |  |  |
| Variable | OR (95% CI) / β (95% HDI) | p-value/ROPE |
| **Brain MRI metrics** |  |  |
| PCOS | 0.0444 (-0.0588, 0.95) | 0.87% |
| Hippocampus GMVol | 0.0269 (-0.0213, 0.95) | 1.00% |
| PCOS × Hippocampus GMVol | -0.0486 (-0.1506, 0.95) | 0.87% |
|  |  |  |
| PCOS | 0.0433 (-0.0601, 0.95) | 0.88% |
| Amygdala GMVol | -0.0213 (-0.0697, 0.95) | 1.00% |
| PCOS × Amygdala GMVol | -0.0318 (-0.1282, 0.95) | 0.94% |
|  |  |  |
| PCOS | 0.0431 (-0.0629, 0.95) | 0.88% |
| FMC GMVol | -0.0169 (-0.0620, 0.95) | 1.00% |
| PCOS × FMC GMVol | 0.0893 (-0.0094, 0.95) | 0.58% |
|  |  |  |
| PCOS | 0.0526 (-0.0504, 0.95) | 0.84% |
| FOC GMVol | -0.0466 (-0.0933, 0.95) | 1.00% |
| PCOS × FOC GMVol | 0.1817 (0.0804, 0.95) * | 0.03% |
|  |  |  |
| PCOS | 0.0499 (-0.0506, 0.95) | 0.85% |
| Accumbens Vol | -0.0205 (-0.0683, 0.95) | 1.00% |
| PCOS × Accumbens Vol | 0.0916 (-0.0050, 0.95) | 0.57% |
|  |  |  |
| PCOS | 0.0426 (-0.0603, 0.95) | 0.89% |
| Thalamus Vol | 0.0245 (-0.0237, 0.95) | 1.00% |
| PCOS × Thalamus Vol | -0.0136 (-0.1101, 0.95) | 0.98% |
|  |  |  |
| PCOS | 0.0434 (-0.0543, 0.95) | 0.87% |
| RostAntCing Thk | 0.0011 (-0.0484, 0.95) | 1.00% |
| PCOS × RostAntCing Thk | -0.0015 (-0.0964, 0.95) | 1.00% |
|  |  |  |
| PCOS | 0.0444 (-0.0602, 0.95) | 0.88% |
| CaudalAntCing Thk | -0.0685 (-0.1131, 0.95) | 0.93% |
| PCOS × CaudalAntCing Thk | 0.1205 (0.0160, 0.95) * | 0.36% |
|  |  |  |
| PCOS | 0.0416 (-0.0603, 0.95) | 0.88% |
| Insula Thk | -0.0500 (-0.1001, 0.95) | 1.00% |
| PCOS × Insula Thk | 0.0801 (-0.0143, 0.95) | 0.67% |
|  |  |  |
| PCOS | 0.0408 (-0.0632, 0.95) | 0.88% |
| MedOrbFront Thk | -0.0208 (-0.0723, 0.95) | 1.00% |
| PCOS × MedOrbFront Thk | 0.0458 (-0.0549, 0.95) | 0.88% |
|  |  |  |
| PCOS | 0.0415 (-0.0638, 0.95) | 0.88% |
| LatOrbFront Thk | -0.0238 (-0.0708, 0.95) | 1.00% |
| PCOS × LatOrbFront Thk | 0.0561 (-0.0506, 0.95) | 0.80% |
|  |  |  |
| PCOS | 0.0261 (-0.0708, 0.95) | 0.95% |
| CH FA | 0.0075 (-0.0377, 0.95) | 1.00% |
| PCOS × CH FA | -0.1051 (-0.2012, 0.95) | 0.46% |
|  |  |  |
| PCOS | 0.0201 (-0.0803, 0.95) | 0.96% |
| CH ICVF | -0.0085 (-0.0534, 0.95) | 1.00% |
| PCOS × CH ICVF | -0.0713 (-0.1799, 0.95) | 0.71% |
|  |  |  |
| PCOS | 0.0330 (-0.0677, 0.95) | 0.92% |
| CCG FA | -0.0036 (-0.0462, 0.95) | 1.00% |
| PCOS × CCG FA | 0.0315 (-0.0866, 0.95) | 0.89% |
|  |  |  |
| PCOS | 0.0302 (-0.0680, 0.95) | 0.93% |
| CCG ICVF | -0.0313 (-0.0763, 0.95) | 1.00% |
| PCOS × CCG ICVF | 0.0101 (-0.1057, 0.95) | 0.96% |
|  |  |  |
| PCOS | 0.0366 (-0.0661, 0.95) | 0.90% |
| UF FA | -0.0255 (-0.0706, 0.95) | 1.00% |
| PCOS × UF FA | 0.0496 (-0.0499, 0.95) | 0.86% |
|  |  |  |
| PCOS | 0.0331 (-0.0682, 0.95) | 0.92% |
| UF ICVF | -0.0204 (-0.0675, 0.95) | 1.00% |
| PCOS × UF ICVF | 0.0265 (-0.0842, 0.95) | 0.93% |
|  |  |  |
| PCOS | 0.0127 (-0.0918, 0.95) | 0.97% |
| Amygdala BOLD Effect | 0.0020 (-0.0475, 0.95) | 1.00% |
| PCOS × Amygdala BOLD Effect | 0.0177 (-0.0787, 0.95) | 0.97% |
|  |  |  |
| In the interactive analyses of brain MRI metrics, data are presented as beta (β) coefficients (posterior medians) with 95% highest density intervals (HDIs) . * Indicates statistical significance (95% HDI excludes zero). ROPE represents the percentage of the posterior distribution within the Region of Practical Equivalence ([-0.1, 0.1]); values < 2.5% suggest practical significance. All brain metrics were standardized (Z-scores). | | |
