# Supplementary material for: From metabolic network to brain loop: unraveling the neuro-metabolic correlates in PCOS-related depression
Source: Front Endocrinol (Lausanne). 2026 Sep 21;17:1925127. doi: 10.3389/fendo.2026.1925127 (PMC13635201; doi:10.3389/fendo.2026.1925127)
Supplement: Supplementary file 2 [file Table10.docx]

| **Supplementary Table 10. Sensitivity analysis for metformin** | | | |
| --- | --- | --- | --- |
| Variable | t | df | p-value |
| FOC GMVol | 0.049 | 4.08 | 0.963 |
| CaudalAntCing Thk | 0.212 | 4.43 | 0.842 |
| Welch-corrected two-sample t-tests were performed to examine potential confounding effects of metformin use on brain metrics. No significant group differences were observed between metformin users (n = 39) and non-users (n = 2787). Analyses for antidepressants and oral contraceptives were not feasible, because UK Biobank field-20003 contains diverse product-level SNOMED-CT codes that cannot be reliably aggregated to define unambiguous medication-use status, which may introduce residual confounding. | | | |
