# Supplementary material for: From metabolic network to brain loop: unraveling the neuro-metabolic correlates in PCOS-related depression
Source: Front Endocrinol (Lausanne). 2026 Sep 21;17:1925127. doi: 10.3389/fendo.2026.1925127 (PMC13635201; doi:10.3389/fendo.2026.1925127)
Supplement: Supplementary file 3 [file Table2.docx]

| Supplementary Table 2. Detailed Mediating Effects of Brain MRI Metrics in PCOS-Related Depression | | | |
| --- | --- | --- | --- |
|  |  |  |  |
| Mediator and Path | β (95% HDIs) | Pd (%) | Mediation Effect |
|  |  |  |  |
| Brain MRI metrics |  |  |  |
| PCOS→Hippocampus GMVol | -0.0302 (-0.2273 ~ 0.1669) | 38.76% |  |
| Hippocampus GMVol→depression | 0.0199 (-0.0357 ~ 0.0755) | 76.12% |  |
| PCOS→Hippocampus GMVol→depression | -0.0006 (-0.0085 ~ 0.0073) | 44.16% | Not Significant |
|  |  |  |  |
| PCOS→Amygdala GMVol | -0.0338 (-0.2045 ~ 0.1369) | 35.75% |  |
| Amygdala GMVol→depression | -0.0449 (-0.1101 ~ 0.0203) | 9.51% |  |
| PCOS→Amygdala GMVol→depression | 0.0015 (-0.0081 ~ 0.0111) | 61.21% | Not Significant |
|  |  |  |  |
| PCOS→FMC GMVol | 0.0452 (-0.1845 ~ 0.2749) | 65.04% |  |
| FMC GMVol→depression | 0.0029 (-0.0485 ~ 0.0543) | 54.77% |  |
| PCOS→FMC GMVol→depression | 0.0001 (-0.0071 ~ 0.0073) | 51.49% | Not Significant |
|  |  |  |  |
| PCOS→FOC GMVol | -0.0549 (-0.2243 ~ 0.1145) | 26.54% |  |
| FOC GMVol→depression | -0.0163 (-0.0871 ~ 0.0545) | 31.23% |  |
| PCOS→FOC GMVol→depression | 0.0009 (-0.0063 ~ 0.0081) | 59.01% | Not Significant |
|  |  |  |  |
| PCOS→Accumbens Vol | -0.0880 (-0.2974 ~ 0.1214) | 20.03% |  |
| Accumbens Vol→depression | 0.0046 (-0.0506 ~ 0.0600) | 56.45% |  |
| PCOS→Accumbens Vol→depression | -0.0004 (-0.0090 ~ 0.0082) | 46.23% | Not Significant |
|  |  |  |  |
| PCOS→Thalamus Vol | 0.0280 (-0.1160 ~ 0.1720) | 64.75% |  |
| Thalamus Vol→depression | 0.0401 (-0.0375 ~ 0.1177) | 83.55% |  |
| PCOS→Thalamus Vol→depression | 0.0011 (-0.0070 ~ 0.0092) | 60.22% | Not Significant |
|  |  |  |  |
| PCOS→RostAntCing Thk | 0.0361 (-0.0041 ~ 0.1941) | 96.60% |  |
| RostAntCing Thk→depression | 0.0088 (-0.2845 ~ 0.3021) | 52.80% |  |
| PCOS→RostAntCing Thk→depression | 0.0004 (-0.0129 ~ 0.0137) | 52.50% | Not Significant |
|  |  |  |  |
| PCOS→CaudalAntCing Thk | 0.0039 (-0.0670 ~ 0.1739) | 53.99% |  |
| CaudalAntCing Thk→depression | -0.1834 (-0.3481 ~ 0.0213) | 1.41% |  |
| PCOS→CaudalAntCing Thk→depression | -0.0007 (-0.0148 ~ 0.0134) | 46.20% | Not Significant |
|  |  |  |  |
| PCOS→Insula_Thk | 0.0056 (-0.0316 ~ 0.1916) | 62.01% |  |
| Insula Thk→depression | -0.2167 (-0.5275 ~ 0.0941) | 8.92% |  |
| PCOS→Insula Thk→depression | -0.0011 (-0.0129 ~ 0.0107) | 40.09% | Not Significant |
|  |  |  |  |
| PCOS→MedOrbFront Thk | 0.0303 (-0.0072 ~ 0.1872) | 93.84% |  |
| MedOrbFront Thk→depression | -0.0587 (-0.3554 ~ 0.2380) | 35.00% |  |
| PCOS→MedOrbFront Thk→depression | -0.0018 (-0.0138 ~ 0.0102) | 37.26% | Not Significant |
|  |  |  |  |
| PCOS→LatOrbFront Thk | 0.0132 (-0.0158 ~ 0.1838) | 79.99% |  |
| LatOrbFront Thk→depression | -0.1149 (-0.4798 ~ 0.2499) | 26.45% |  |
| PCOS→LatOrbFront Thk→depression | -0.0015 (-0.0113 ~ 0.0083) | 35.84% | Not Significant |
|  |  |  |  |
| PCOS→CH FA | -0.0001 (-0.0084 ~ 0.0082) | 48.60% |  |
| CH FA→depression | -0.4486 (-1.6928 ~ 0.7956) | 24.68% |  |
| PCOS→CH FA→depression | 0.0000 (-0.0073 ~ 0.0073) | 50.70% | Not Significant |
|  |  |  |  |
| PCOS→CH ICVF | -0.0033 (-0.0110 ~ 0.0044) | 20.12% |  |
| CH ICVF→depression | -0.6549 (-1.9886 ~ 0.6788) | 17.82% |  |
| PCOS→CH ICVF→depression | 0.0022 (-0.0058 ~ 0.0102) | 68.47% | Not Significant |
|  |  |  |  |
| PCOS→CCG FA | -0.0005 (-0.0087 ~ 0.0077) | 45.55% |  |
| CCG FA→depression | 0.0134 (-1.2566 ~ 1.2834) | 50.31% |  |
| PCOS→CCG FA→depression | 0.0000 (-0.0060 ~ 0.0060) | 49.81% | Not Significant |
|  |  |  |  |
| PCOS→CCG ICVF | -0.0031 (-0.0125 ~ 0.0063) | 26.20% |  |
| CCG ICVF→depression | -0.8070 (-1.9066 ~ 0.2926) | 8.29% |  |
| PCOS→CCG ICVF→depression | 0.0025 (-0.0076 ~ 0.0126) | 69.74% | Not Significant |
|  |  |  |  |
| PCOS→UF FA | -0.0076 (-0.0168 ~ 0.0016) | 5.73% |  |
| UF FA→depression | -0.4267 (-1.5457 ~ 0.6923) | 23.77% |  |
| PCOS→UF FA→depression | 0.0033 (-0.0070 ~ 0.0136) | 73.22% | Not Significant |
|  |  |  |  |
| PCOS→UF ICVF | -0.0041 (-0.0115 ~ 0.0033) | 14.22% |  |
| UF ICVF→depression | -0.5311 (-1.9715 ~ 0.9093) | 23.72% |  |
| PCOS→UF ICVF→depression | 0.0022 (-0.0059 ~ 0.0103) | 68.88% | Not Significant |
|  |  |  |  |
| PCOS→amygdala BOLD effect | -0.0301 (-0.0767 ~ 0.0165) | 11.34% |  |
| amygdala BOLD effect→depression | -0.0061 (-0.2934 ~ 0.2812) | 47.96% |  |
| PCOS→amygdala BOLD effect→depression | 0.0002 (-0.0115 ~ 0.0119) | 52.08% | Not Significant |
|  |  |  |  |
| Brain MRI Metrics: Analyses were performed using Bayesian mediation models. Data are presented as posterior medians (β) with 95% highest density intervals (HDIs). Statistical significance was determined by the Probability of Direction (Pd > 97.5%).  Values in the Mediation effect represent the proportion of the total effect mediated. A negative value indicates a suppression effect. | | | |
