# Supplementary material for: From metabolic network to brain loop: unraveling the neuro-metabolic correlates in PCOS-related depression
Source: Front Endocrinol (Lausanne). 2026 Sep 21;17:1925127. doi: 10.3389/fendo.2026.1925127 (PMC13635201; doi:10.3389/fendo.2026.1925127)
Supplement: Supplementary file 4 [file Table3.docx]

| Supplementary Table 3. Relative Weight, ΔR² and PCA. | | | | | |
| --- | --- | --- | --- | --- | --- |
| Variable | LWG Relative Weight | | PCA Loadings | | |
|  | Weight (%) | ΔR² (%) | PC1 (Metabolic) | PC2  (Brain MRI) | PC3 (Inflammation) |
| **PCOS status** |  |  |  |  |  |
| PCOS | 0.28 | 0.03 | - | - | - |
| **Basic covariates** |  |  |  |  |  |
| 7 basic covariates | 64.29 | 7.76 | - | - | - |
| **Metabolic biomarkers** |  |  |  |  |  |
| BMI | 0.29 | 0.02 | 0.59 * | 0.16 | -0.28 |
| FAI | 2.28 | 0.31 | 0.53 * | 0.04 | -0.15 |
| TyG | 2.33 | 0.35 | 0.53 * | 0.28 | 0.21 |
| SII | 9.70 | 0.88 | 0.18 | -0.15 | 0.87 * |
| **Brain MRI metrics** |  |  |  |  |  |
| FOC GMVol | 0.84 | 0.10 | -0.16 | 0.66 * | 0.29 |
| CaudalAntCing Thk | 19.49 | 1.99 | -0.20 | 0.66 * | -0.11 |
|  |  |  |  |  |  |
| Full model weighted R² (%) |  | 10.89 |  |  |  |
| Variance Explained (%) |  |  | 26.80 | 19.20 | 17.40 |
| Relative weights were calculated using the Lindeman–Merenda–Gold (LMG) method.  ΔR² represents the independent incremental explanatory power of each domain after adjustment for all other variables, calculated as the difference in weighted R² between the full model and nested models excluding the corresponding category.  Sum of relative weights for all 7 basic covariates including age, ethnicity, education, physical activity, smoking, alcohol, menopause.  PCA loadings with absolute value > 0.5 indicated strong contributions and were highlighted with *.  Variance explained indicates the proportion of total variance in the six indicators captured by each PC. | | | | | |
