# Supplementary material for: From metabolic network to brain loop: unraveling the neuro-metabolic correlates in PCOS-related depression
Source: Front Endocrinol (Lausanne). 2026 Sep 21;17:1925127. doi: 10.3389/fendo.2026.1925127 (PMC13635201; doi:10.3389/fendo.2026.1925127)
Supplement: Supplementary file 5 [file Table4.docx]

| Supplemental Table 4: Eigenvalues, Variance Explained and Variable Loadings of Principal Components | | | | | | | |
| --- | --- | --- | --- | --- | --- | --- | --- |
|  |  |  |  |  |  |  |  |
| Variable | PC1 | PC2 | PC3 | PC4 | PC5 | PC6 | Communality |
| BMI | 0.586 * | 0.164 | -0.275 | - | - | - | 0.72 |
| FAI | 0.529 * | 0.036 | -0.149 | - | - | - | 0.68 |
| TyG | 0.528 * | 0.282 | 0.205 | - | - | - | 0.71 |
| SII | 0.178 | -0.149 | 0.873 * | - | - | - | 0.85 |
| FOC | -0.157 | 0.662 * | 0.294 | - | - | - | 0.69 |
| Caudal ACC | -0.204 | 0.657 * | -0.11 | - | - | - | 0.73 |
| Eigenvalue | 1.61 | 1.152 | 1.045 | 0.849 | 0.765 | 0.579 |  |
| Variance (%) | 26.84 | 19.2 | 17.41 | 14.16 | 12.75 | 9.65 |  |
| Cumulative % | 26.84 | 46.04 | 63.45 | 77.61 | 90.35 | 100 |  |
|  |  |  |  |  |  |  |  |
| Bartlett's test of sphericity was significant (χ² = 77.60, p < 0.001), indicating that the six variables were sufficiently correlated to perform principal component analysis. Components with eigenvalues greater than 1 were retained for further interpretation.  Loadings with absolute values greater than 0.5 indicated strong contributions to the respective principal components *, which is a commonly used empirical threshold in PCA studies to clarify the core variables of each component. | | | | | | | |
