# Supplementary material for: From metabolic network to brain loop: unraveling the neuro-metabolic correlates in PCOS-related depression
Source: Front Endocrinol (Lausanne). 2026 Sep 21;17:1925127. doi: 10.3389/fendo.2026.1925127 (PMC13635201; doi:10.3389/fendo.2026.1925127)
Supplement: Supplementary file 6 [file Table5.docx]

| Supplementary Table 5. Dose-Dependent and Linearity Analyses of Metabolic Biomarkers | | | | |
| --- | --- | --- | --- | --- |
| Groups | Dose-Response Analysis | | Continuous Trend Nonlinear Test (GAM) | |
|  | OR (95% CI) | p-value | edf | p-value |
| BMI |  |  |  |  |
| Overweight vs. Normal | 1.442 (1.021-2.033) | 0.038 * |  |  |
| Obesity vs. Normal | 1.552 (0.983-2.451) | 0.058 † | 1.000898 | 0.004 ** |
|  |  |  |  |  |
| FAI |  |  |  |  |
| Hyperandrogenism vs. Normoandrogenism | 0.781 (0.424-1.432) | 0.420 | 1.00058 | 0.040 * |
|  |  |  |  |  |
| SII |  |  |  |  |
| High inflammation vs. Low inflammation | 1.542 (1.161-2.044) | 0.003 ** | 1.000314 | 0.000 ** |
|  |  |  |  |  |
| TyG |  |  |  |  |
| Insulin resistance vs.Non-insulin resistance | 1.283 (0.931-1.762) | 0.134 | 1.000772 | 0.063 † |
| Dose-response analysis: results were presented as OR with 95% CI and p-values. OR > 1 indicates increased depression risk, while OR < 1 indicates decreased risk. Continuous Trend Nonlinear Test (GAM): edf > 1 suggested a nonlinear trend, edf ≈ 1 suggested a near-linear trend, p-values indicated the statistical significance of the trend. Statistical significance was defined as * p < 0.05, ** p < 0.01. A marginally trend was defined as † p-value close to 0.05. | | | | |
