# Supplementary material for: From metabolic network to brain loop: unraveling the neuro-metabolic correlates in PCOS-related depression
Source: Front Endocrinol (Lausanne). 2026 Sep 21;17:1925127. doi: 10.3389/fendo.2026.1925127 (PMC13635201; doi:10.3389/fendo.2026.1925127)
Supplement: Supplementary file 7 [file Table6.docx]

| Supplementary Table 6. Mediating Effects of Metabolic Biomarkers Disappeared in the PCOS-Depression After Adjusting for Brain MRI Metrics | | |
| --- | --- | --- |
| Mediator and Path | β (95% CI) | p-value |
| **Total Sample** |  |  |
| PCOS→BMI | 0.3523 (-0.0454 ~ 0.7466) | Not significant |
| BMI→depression | -0.0518 (-0.5113 ~ 0.3606) | Not significant |
| PCOS→BMI→depression | -0.0183 (-0.2395 ~ 0.1467) | Not significant |
|  |  |  |
| PCOS→FAI | 0.3577 (-0.0199 ~ 0.8079) | Not significant |
| FAI→depression | 0.1003 (-0.2907 ~ 0.5391) | Not significant |
| PCOS→FAI→depression | 0.0359 (-0.1512 ~ 0.2000) | Not significant |
|  |  |  |
| **Normal BMI Subgroup** |  |  |
| PCOS→BMI | 0.0579 (-0.1415, 0.2787) | Not significant |
| BMI→depression | 0.1245 (-1.8570, 2.5575) | Not significant |
| PCOS→BMI→depression | 0.0072 (-0.2875, 0.3359) | Not significant |
|  |  |  |
| PCOS→FAI | 0.2189 (-0.1409, 0.5170) | Not significant |
| FAI→depression | 0.6875 (-0.0362, 2.2094) | Not significant |
| PCOS→FAI→depression | 0.1505 (-0.1576, 0.6502) | Not significant |
|  |  |  |
| **Overweight/Obesity Subgroup** |  |  |
| PCOS→BMI | 0.3675 (-0.1091, 0.7870) | Not significant |
| BMI→depression | -0.0509 (-1.2965, 0.9370) | Not significant |
| PCOS→BMI→depression | -0.0187 (-0.6218, 0.3085) | Not significant |
|  |  |  |
| PCOS→FAI | 0.3707 (-0.2832, 1.0856) | Not significant |
| FAI→depression | -0.0373 (-1.0558, 0.8254) | Not significant |
| PCOS→FAI→depression | -0.0138 (-0.7015, 0.2948) | Not significant |
|  |  |  |
| **Low FAI Subgroup (≤ median)** |  |  |
| PCOS→BMI | 0.3281 (-0.3710, 1.0146) | Not significant |
| BMI→depression | 0.9454 (-0.0209, 101.7058) | Not significant |
| PCOS→BMI→depression | 0.3102 (-1.0612, 12.1879) | Not significant |
|  |  |  |
| PCOS→FAI | -0.0858 (-0.2971, 0.1249) | Not significant |
| FAI→depression | -0.3664 (-6.1822, 6.6157) | Not significant |
| PCOS→FAI→depression | 0.0314 (-0.6185, 1.1071) | Not significant |
|  |  |  |
| **High FAI Subgroup (> median)** |  |  |
| PCOS→BMI | 0.1376 (-0.3488, 0.6305) | Not significant |
| BMI→depression | -0.4397 (-1.7198, 0.1313) | Not significant |
| PCOS→BMI→depression | -0.0605 (-0.6109, 0.2347) | Not significant |
|  |  |  |
| PCOS→FAI | 0.1454 (-0.4045, 0.7435) | Not significant |
| FAI→depression | -0.2820 (-1.4038, 0.3606) | Not significant |
| PCOS→FAI→depression | -0.0410 (-0.6379, 0.1631) | Not significant |
| Data were presented as β with 95% CIs. * A 95% CI not crossing zero was considered statistically significant. * p-value <0.05 was considered statistically significant. A marginally significance was defined as † p=0.05. A negative value indicates a suppression effect. | | |
