# Supplementary material for: From metabolic network to brain loop: unraveling the neuro-metabolic correlates in PCOS-related depression
Source: Front Endocrinol (Lausanne). 2026 Sep 21;17:1925127. doi: 10.3389/fendo.2026.1925127 (PMC13635201; doi:10.3389/fendo.2026.1925127)
Supplement: Supplementary file 8 [file Table7.docx]

| Supplementary Table 7. The Independent Interaction Effects of Brain MRI Metrics in PCOS-Related Depression | | |
| --- | --- | --- |
| Variable | Estimate (95% CI) | Pr(>\|t\|) |
| PCOS | 0.005 (0.000 ~ 0.010) | 0.940 |
| FOC GMVol | -0.041 (-0.081 ~ 0.000) | 0.141 |
| PCOS × FOC GMVol | 0.154 (0.029 ~ 0.279) | 0.016 * |
|  |  |  |
| PCOS | 0.009 (0.000 ~ 0.018) | 0.891 |
| CaudalAntCing Thk | -0.076 (-0.150 ~ 0.000) | 0.005 ** |
| PCOS × CaudalAntCing Thk | 0.145 (0.012 ~ 0.279) | 0.034 * |
|  |  |  |
| PCOS | 0.050 (0.000 ~ 0.100) | 0.458 |
| BMI | 0.002 (-0.047 ~ 0.051) | 0.940 |
| PCOS × BMI | -0.064 (-0.125 ~ 0.002) | 0.249 |
|  |  |  |
| PCOS | 0.029 (0.000 ~ 0.057) | 0.671 |
| FAI | 0.019 (-0.028 ~ 0.066) | 0.442 |
| PCOS × FAI | -0.011 (-0.033~ 0.011) | 0.829 |
|  |  |  |
| PCOS | 0.022 (0.000 ~ 0.044) | 0.738 |
| SII | 0.033 (0.000 ~ 0.067) | 0.177 |
| PCOS × SII | 0.053 (0.000 ~ 0.106) | 0.431 |
|  |  |  |
| PCOS | 0.033 (0.000 ~ 0.065) | 0.620 |
| TyG | 0.025 (-0.024 ~ 0.074) | 0.331 |
| PCOS × TyG | -0.005 (-0.016 ~ 0.005) | 0.936 |
| Data were analyzed using weighted linear regression models with sampling weights for 1:5 age matching. All continuous variables were standardized to z-scores. Estimate represented the regression coefficient of the interaction term, and Pr(>\|t\|) < 0.05 was defined as statistical significance. | | |
