# Supplementary material for: From metabolic network to brain loop: unraveling the neuro-metabolic correlates in PCOS-related depression
Source: Front Endocrinol (Lausanne). 2026 Sep 21;17:1925127. doi: 10.3389/fendo.2026.1925127 (PMC13635201; doi:10.3389/fendo.2026.1925127)
Supplement: Supplementary file 9 [file Table8.docx]

| Supplementary Table 8. The Association Between FOC GMVol and CaudalAntCing Thk Existed in Non-PCOS Individuals | | | | | |
| --- | --- | --- | --- | --- | --- |
| Subgroup | Mediator and Path | Unadjusted for Metabolic Biomarkers | | Adjusted for Metabolic Biomarkers | |
|  |  | β / OR (95% CI) | p-value | β / OR (95% CI) | p-value |
| Total  Sample | FOC GMVol→CaudalAntCing Thk | 0.124 (0.014~0.235) | 0.028 * | 0.129 (0.001~0.257) | 0.048 * |
|  | CaudalAntCing Thk→Depression | 0.788 (0.576~1.079) | 0.137 | 0.663（0.465~0.945） | 0.023 * |
|  | FOC GMVol→CaudalAntCing Thk→Depression | - | - | 0.947 (0.844~1.002) | 0.441 |
|  | CaudalAntCing Thk→FOC GMVol | 0.129 (0.015~0.242) | 0.028 * | 0.129 (0.001~0.257) | 0.048 * |
|  | FOC GMVol→Depression | 0.938 (0.686~1.285) | 0.692 | 0.898（0.633~1.272） | 0.544 |
|  | CaudalAntCing Thk→FOC GMVol→Depression | - | - | - | - |
|  |  |  |  |  |  |
| PCOS | FOC GMVol→CaudalAntCing Thk | 0.049 (-0.047~0.145) | 0.307 | 0.053 (-0.256~0.362) | 0.740 |
|  | CaudalAntCing Thk→Depression | 13.166 (0.215~1842.313) | 0.251 | 1.200 (0.272~5.292) | 0.810 |
|  | FOC GMVol→CaudalAntCing Thk→Depression | - | - | - | - |
|  | CaudalAntCing Thk→FOC GMVol | 0.391 (-0.369~1.151) | 0.307 | 0.062 (-0.303~0.427) | 0.740 |
|  | FOC GMVol→Depression | 4.601 (1.245~23.234) | 0.036 * | 1.329 (0.464~3.806) | 0.596 |
|  | CaudalAntCing Thk→FOC GMVol→Depression | - | - | - | - |
|  |  |  |  |  |  |
| non-PCOS | FOC GMVol→CaudalAntCing Thk | 0.054 (0.002~0.106) | 0.042 * | 0.152 (0.005~0.299) | 0.045 * |
|  | CaudalAntCing Thk→Depression | 0.258 (0.061~1.051) | 0.060 † | 0.612 (0.413~0.908) | 0.015 * |
|  | FOC GMVol→CaudalAntCing Thk→Depression | - | - | 0.936 (0.814~0.999) | 0.455 |
|  | CaudalAntCing Thk→FOC GMVol | 0.325 (0.011~0.638) | 0.042 * | 0.145 (0.004~0.286) | 0.045 * |
|  | FOC GMVol→Depression | 0.549 (0.287~1.009) | 0.060 † | 0.707 (0.464~1.078) | 0.107 |
|  | CaudalAntCing Thk→FOC GMVol→Depression | - | - | - | - |
| Data were presented as β / OR with 95% CI. * p-value <0.05 and 95% CI excludes zero were considered statistically significant. A marginal trend was defined as † p-value close to 0.05. | | | | | |
