# Supplementary material for: From metabolic network to brain loop: unraveling the neuro-metabolic correlates in PCOS-related depression
Source: Front Endocrinol (Lausanne). 2026 Sep 21;17:1925127. doi: 10.3389/fendo.2026.1925127 (PMC13635201; doi:10.3389/fendo.2026.1925127)
Supplement: Supplementary file 10 [file Table9.docx]

| Supplementary Table 9. Interaction Analyses between FOC GMVol and CaudalAntCing Thk and Stratified by PCOS | | | |
| --- | --- | --- | --- |
| Subgroup | Variable | OR (95% CI) | p-value |
| Total | FOC GMVol | 0.94 (0.56–1.57) | 0.8230 |
|  | CaudalAntCing Thk | 0.39 (0.11–1.43) | 0.1520 |
|  | FOC × Caudal Interaction | 0.84 (0.11–6.80) | 0.8700 |
|  |  |  |  |
| PCOS | FOC GMVol | 3.43 (1.29–9.99) | 0.0360 * |
|  | CaudalAntCing Thk | 1.56 (0.29–8.70) | 0.2310 |
|  | FOC × Caudal Interaction | 1.03 (0.17–6.27) | 0.5510 |
|  |  |  |  |
| non-PCOS | FOC GMVol | 0.57 (0.31–1.03) | 0.0850 |
|  | CaudalAntCing Thk | 0.22 (0.05–0.98) | 0.0500 † |
|  | FOC × Caudal Interaction | 0.46 (0.04–5.72) | 0.5330 |
|  |  |  |  |
| Data are presented as OR with 95% CI. * p-value <0.05 was considered statistically significant. A marginally significance was defined as † p-value close to 0.05. | | | |
